# Single-cell transcriptome analysis of bronchoalveolar lavage during early SARS-CoV-2 infection

**DOI:** 10.1128/spectrum.02715-24

**Published:** 2025-07-31

**Authors:** Sadia Akter, Mushtaq Ahmed, Dhiraj K. Singh, Kuldeep S. Chauhan, Deepak Kaushal, Shabaana A. Khader

**Affiliations:** 1 Department of Microbiology, The University of Chicago 456539, Chicago, Illinois, USA; 2 Southwest National Primate Research Center and Host Pathogen Interactions Program, Texas Biomedical Research Institute 7075, San Antonio, Texas, USA; Geisel School of Medicine at Dartmouth, Lebanon, New Hampshire, USA

**Keywords:** COVID-19, SARS-CoV-2, single-cell RNA-seq, myeloid, interferon, interleukins

## Abstract

The COVID-19 pandemic, triggered by the SARS-CoV-2 virus, has led to global consequences since its emergence in late 2019, impacting health, economies, and societies worldwide. Understanding the early responses following infection will provide crucial insights into the immune mechanisms governing disease progression and protection. We employed the single-cell RNA-sequencing approach using bronchoalveolar lavage cells of rhesus macaques at 0–3 days post-infection (dpi) in a non-human primate model of SARS-CoV-2. Our results show the increased abundance of two subsets of macrophages that express IFN-responsive gene signatures, inflammatory dendritic cells (DCs), plasmacytoid DCs, and conventional DCs at 3 dpi, coinciding with peak viremia. Moreover, our investigation underscores the enrichment of IFN-α/β, IFN-γ, and IL-1 signaling pathways within myeloid populations at 2–3 dpi in comparison to baseline. These findings substantially deepen our comprehension, elucidating the transcriptional landscape at the single-cell level during the early phases of COVID-19 within a highly relevant model.

**IMPORTANCE:**

Understanding the immune response in the early stages of COVID-19 is crucial for developing better treatments and vaccines. In this study, we used advanced single-cell RNA sequencing to examine the immune cells in the lungs of rhesus macaques, a model for human SARS-CoV-2 infection, within the first few days after infection. We identify key immune cell types, such as macrophages and dendritic cells, that are activated early on and produce important signals to fight the virus. These findings help clarify how the immune system responds to SARS-CoV-2 at the single-cell level, offering valuable insights that could guide future therapeutic approaches.

## INTRODUCTION

Since its emergence in late 2019, the novel coronavirus SARS-CoV-2, responsible for the COVID-19 pandemic, has created unprecedented challenges in the world. The rapid spread and high morbidity and mortality rates associated with COVID-19 have necessitated an urgent need for a comprehensive understanding of the pathogenesis of the disease at a molecular and cellular level. A fundamental aspect of strengthening our knowledge about COVID-19 involves investigating the interactions between the virus and host cells.

The main causes of mortality in COVID-19 patients are extensive multi-organ dysfunction, acute respiratory distress syndrome (ARDS), cardiac failure, and renal collapse, which are accompanied by dysregulated immunological signatures (1). The dysregulated host immune response results in the production of an inflammatory cytokine storm, presented in a subset of patients with severe COVID-19. Alterations in gene expression within cells from the bronchoalveolar lavage (BAL) and peripheral blood mononuclear cells in patients with COVID-19 have helped identify unique inflammatory cytokine profiles, providing valuable insights into the pathogenesis of the disease (2). Understanding the early molecular and cellular pathways triggered by SARS-CoV-2 infection is essential for developing targeted therapeutic interventions and improving patient outcomes.

Comprehensively exploring the host’s transcriptomic landscape is invaluable for uncovering protective immune mechanisms. Understanding such mechanisms is pivotal in developing control strategies for infection and disease progression. In studies focusing on single cells, the examination predominantly hinges on unique gene expression patterns within immune cells. Single-cell RNA-sequencing (scRNA-seq) has become instrumental in this pursuit, providing an exceptional level of detail in unraveling the heterogeneity of cellular responses to SARS-CoV-2 infection. Through its capability to scrutinize individual cell types within complex tissues, scRNA-seq sheds light on the mechanisms of infection and host immune responses and assists in pinpointing potential therapeutic avenues.

Previous investigations employing scRNA-seq have provided valuable insights into multiple facets of COVID-19. These include highlighting cell types involved in SARS-CoV-2 infection (3), understanding the dynamics of the immune response (4), and uncovering the molecular pathways disrupted by the virus (3–6). Despite these advancements, gaps persist in our comprehension, notably concerning the variability of clinical manifestations and the underlying immune mechanisms that drive the early stages of disease pathogenesis. The current study aims to leverage the capabilities of scRNA-seq to uncover the initial interaction between SARS-CoV-2 and the host cellular environment. Recently, we established a nonhuman primate (NHP) model for SARS-CoV-2 infection by infecting rhesus macaques, which subsequently display COVID-19 symptoms such as distinct ground glass appearances in lung tissues (7) and coincide with a cytokine storm and an influx of myeloid cells, ultimately leading to viral clearance and recovery (7). An in-depth understanding of the early immune interactions at the single-cell level will provide novel insights into the pathophysiology of COVID-19.

There is limited information on the early molecular changes in host immune response following SARS-CoV-2 infection. Maurya et al.’s study (8) revealed distinct early innate immune responses in various COVID-19 sub-phenotypes, based on transcriptomic analysis of nasopharyngeal samples from patients. Longitudinal assessment of stimulated immune responses in COVID-19 patients revealed that early impairments in innate immune response are associated with subsequent COVID-19 disease severity (9). This study aims to understand the early immune response to SARS-CoV-2 infection in the NHP model, focusing on the critical 0–3-day post-infection (dpi) period. The data revealed that genes linked to interferon, interleukins (IL), and neutrophil degranulation are activated as early as day 2 in the BAL as a response to the virus, providing valuable insights for developing effective clinical intervention strategies and therapeutic approaches.

## RESULTS

### Single-cell profiling of immune landscape during very early stages of SARS-CoV-2 infection

**Fig 1:**
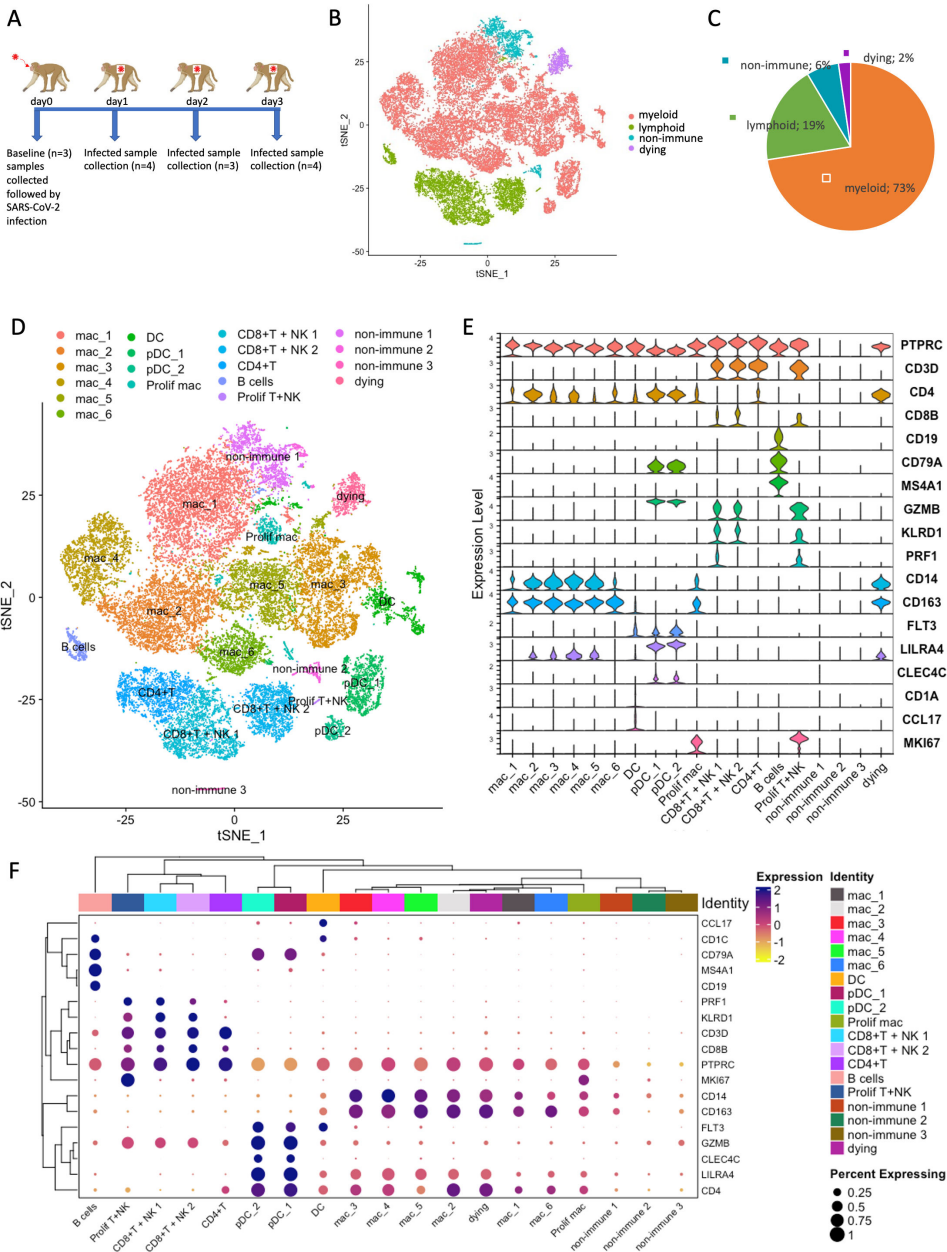
scRNA-seq transcriptional profiling of SARS-CoV-2 infected rhesus macaques. (**A**) Study design using 10× genomics platform. Rhesus macaques were infected with SARS-CoV-2, followed by BAL samples being collected at baseline-infected at day 0 (*n* = 3), infected at 1 dpi (*n* = 4), infected at 2 dpi (*n* = 3), and infected at 3 dpi (*n* = 4). (**B**) t-Distributed stochastic neighbor embedding (tSNE) visualization of the major cell types representing non-immune/lymphoid/myeloid cells in the data set (all conditions together). Colored by cell type. (**C**) Pie charts representing the distribution of major cell types across conditions (baseline, 1 dpi, 2 dpi, and 3 dpi). (**D**) tSNE visualization of major cell sub-types (all conditions together). Colored according to cellular identity. (**E**) Distribution of the expression of known markers in different clusters. (**F**) Dot plot and clustering of the expression of top markers in each cluster. The dot color represents the expression level, and the dot size represents the percentage of cells in each cluster expressing the gene. Similar types of cells/clusters group together by cluster analysis.

To characterize the response of immune cells in the BAL at very early stages of SARS-CoV-2 infection, which are missed by clinical interrogation, we performed 10× scRNA-seq on single cells from BAL of rhesus macaques infected by SARS-CoV-2 at different time points: day 0 (SARS-CoV-2 infection, baseline, *n* = 3), 1 dpi (1 dpi Inf, *n* = 4), 2 dpi (*n* = 3), and 3 dpi (*n* = 4; Fig. 1A).

After demultiplexing, alignment, and quality control process, we found a total of 31,939 cells (214–12,683 cells per sample) that were of high quality and comparable across the samples (Table 1). The average number of cells per sample at baseline (5,869) was higher than 1 dpi (487 cells) and 2 dpi (694 cells per sample) of the infected lungs, which later increased at 3 dpi (2,576 cells per sample; Table 2). Combining all samples, we observed that myeloid cell populations were abundant in the BAL and constituted 73% of the total cell count, compared to 19% for lymphoid cells across all time points, which is consistent with previous studies (10–12). Additionally, non-immune cells added up to 6%, and dying cells made up 2% of the total cells (Fig. 1B and C).

**TABLE 1:** Total cells in each sample

Sample ID	Cell count
AS023_1	2,828
AS023_11	1,140
AS023_12	727
AS023_13	2,617
AS023_14	3,209
AS023_15	2,996
AS023_16	1,481
AS023_2	12,683
AS023_4	2,097
AS023_5	310
AS023_6	353
AS023_7	1,043
AS023_8	241
AS023_9	214

**TABLE 2:** Average number of cells per condition

Conditions	Average cells (approx.)
Baseline	5,869
Day 1	487
Day 2	694
Day 3	2,576

Based on canonical gene expression, we identified 19 distinct clusters representing different types of cells including macrophage cells (*CD14^+^* and *CD163^+^*), dendritic cells (DC; *FLT3*^+^) (11, 13), plasmacytoid DCs (pDC) cells (*CLEC4C^+^*, *FLT3^+^*, *LILRA4^+^*, and *TCF4^+^*) (13–15), proliferating macrophage cluster (*CD163^+^* and *MKI67^+^*), proliferating lymphoid cluster (*CD3D^+^* and *MKI67^+^*), CD4^+^T cells (*CD3D^+^* and *CD4D^+^*), CD8^+^T and NK cells (*CD3D^+^*, *CD8D^+^*, *GZMB^+^*, *PRF1^+^*, and *KLRD1^+^*), and B cells (*CD19^+^*, *MS4A1^+^*, and *CD79A^+^*; Fig. 1D through F; Fig. S1). We also found three clusters of non-immune cells (*PTPRC^+^*) and one mitochondrial gene-enriched dead cell cluster (*ND4^+^*, *COX2^+^*, and *ATP6^+^*). Since the majority of the cells were of myeloid origin in the BAL samples obtained from the infected macaques in this study, we focused on characterizing myeloid populations at the single cell level in the next steps.

### Myeloid cell populations in BAL during early infection

**Fig 2:**
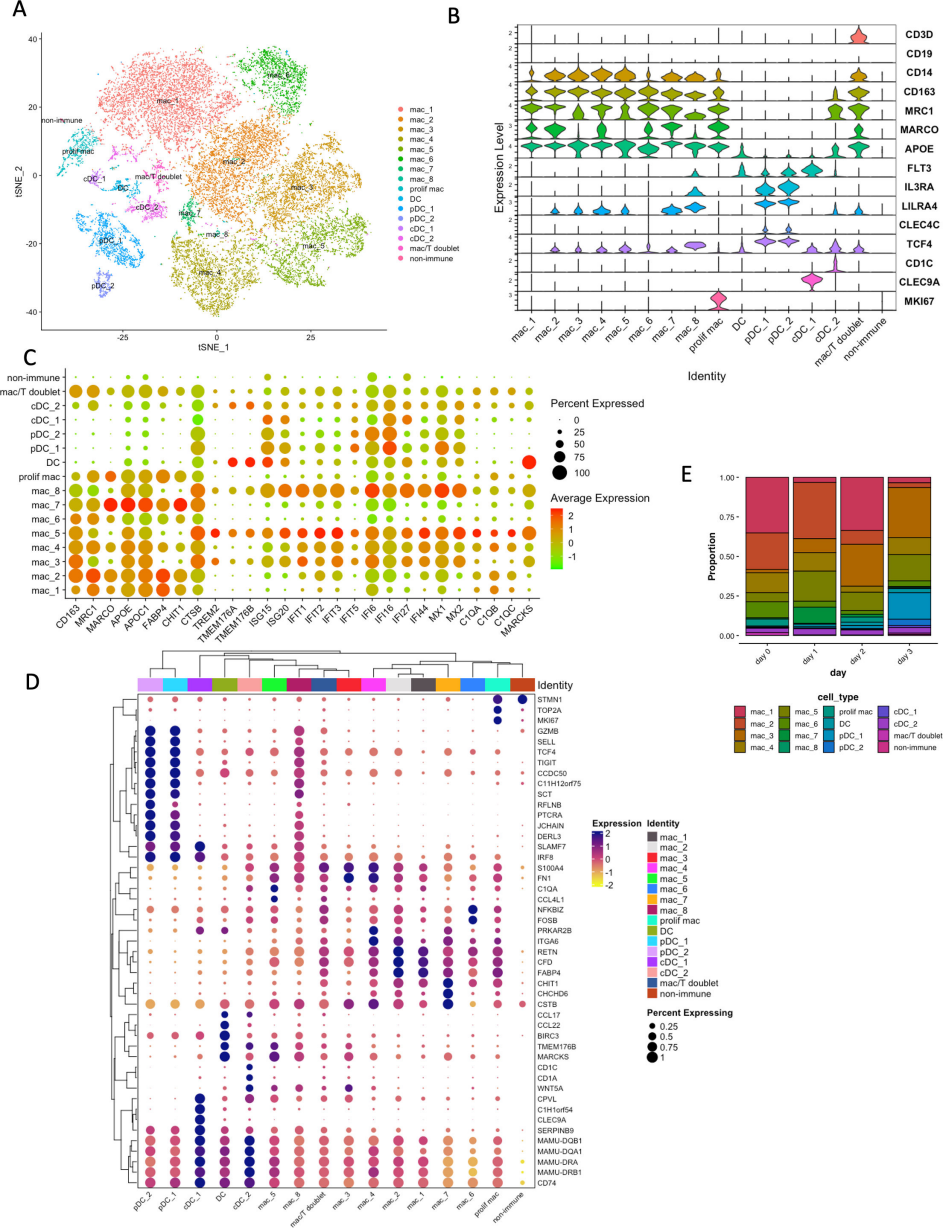
Myeloid cell landscape in the BAL of rhesus macaques. (**A**) t-Distributed stochastic neighbor embedding (tSNE) plot of myeloid cell sub-types after re-clustering the myeloid cells only (all conditions together). Colored according to cellular identity. (**B**) Distribution of the expression of known markers in different myeloid clusters. (**C**) Dot plot of the expression of known myeloid markers and IFN-related genes in different myeloid clusters. The dot color represents the expression level, and the dot size represents the percentage of cells in each cluster expressing the gene. (**D**) Dot plot with clustering of the expression of top markers of different myeloid clusters. The dot color represents the expression level, and the dot size represents the percentage of cells in each cluster expressing the gene. Similar types of cells/clusters grouped together by cluster analysis. (**E**) Bar plot of myeloid cells proportion of each cluster in each condition (baseline, 1 dpi, 2 dpi, and 3 dpi).

We identified 23,161 myeloid cells across all conditions. To better characterize the myeloid populations in BAL, we re-clustered all the myeloid cells. This yielded 16 myeloid sub-populations including eight clusters of macrophages (*CD14^+^* and *CD163^+^*), one proliferating macrophage cluster (*CD14^+^*, *CD163^+^*, and *MKI67^+^*), one inflammatory DC cluster (*FLT3^+^*; DC), two pDC clusters (*CLEC4C^+^*, *FLT3^+^*, *LILRA4^+^*, and *TCF4^+^*) and two conventional DC (cDC) clusters (*CLEC9A^+^/CD1C^+^*; Fig. 2A and B; Fig. S2A). All eight macrophage clusters were CD163^+^ and Mannose Receptor C-Type 1 (*MRC1^+^*), suggesting that they represented alveolar macrophage (AM)-like populations (13) (Fig. 2B; Fig. S2B). Interestingly, at this early stage of infection, we did not identify any MRC1^-^, i.e., interstitial macrophage (IM) populations (7). Our earlier work has identified that IMs are robustly recruited to the lung compartment of SARS-CoV-2-infected macaques at later stages of infection. Recruitment (and turnover) of IMs, which generally have a type I IFN-responsive inflammatory phenotype, is also associated with the progression of disease in macaque models of HIV (16), TB (13), and TB/HIV (17). Thus, very early (0–3 dpi) responses to SARS-CoV-2 infection are dominated by AMs, whereas IMs are recruited later. In addition, these clusters also exhibited other classic markers of AMs, such as MARCO and APOE (Fig. 2B and C; Fig. S2B). Consistent with the previous study, AMs were the major macrophages found in BAL (10).

The first two AM-like macrophage populations—referred to here as *mac_* MRC1^+^FABP4^hi^CHIT1^+^(mac_1) and mac_MRC1^hi^FABP4^hi^CHIT1^+^(mac_2)—highly expressed MRC1, Fatty Acid-Binding Protein 4 (FABP4), which is primarily involved in lipid metabolism and has implications for metabolic diseases and inflammation, and Chitotriosidase 1 (*CHIT1*), which is associated with the immune response and known as a marker for macrophage activation (Fig. 2B), suggesting their contribution to metabolism and immune response. These two clusters were mostly present at the baseline (Fig. 2E; Fig. S2A) and constituted 58.27% of total myeloid cells at baseline in the BALs of macaques.

We identified four AM-like macrophage clusters—annotated as mac_IFN (mac_4), “mac_TREM2^+^IFN” (mac_3), “mac_TREM2^hi^IFN^hi^” (mac_5), and “mac_TREM2^+^IFN^hi^” (mac_8)—that expressed several IFN-responsive gene signatures at moderate to high level, such as interferon induced protein with tetratricopeptide repeats (*IFIT*) *1*, *IFIT2*, *IFIT3*, and *IFIT5*; IFN-α inducible protein 6 (*IFI6*), *IFI16*, and *IFI44*; interferon-stimulated gene (*ISG*) *15* and *ISG 20*; MX dynamin-like GTPase (*MX*) *1* and *MX2*; Complement C1q Chain (*C1Q*) *A*, *B*, and C; C-C Motif Chemokine Ligand 4 Like 1 (*CCL4L1*; Fig. 2C). In addition, the later three clusters expressed moderate to high levels of *TREM2*, Transmembrane Protein 176 (*TMEM176*) A and B, and low levels of *MARCO* (Fig. 2C). The clusters, mac_3 and mac_5, were predominantly present in the BALs of infected macaques at 3 dpi compared to earlier time points, incorporating 31.6% (mac_3) and 16.64% (mac_5) of total myeloid cells at 3 dpi (Fig. 2E; Fig. S2A). We also found two other AM-like clusters, mac_FOSB^+^ (mac_6) and mac_CHIT^hi^ (mac_7). The cluster mac_FOSB^+^ (mac_6) enriched high levels of *FOSB* and *NFKBIZ* and was abundant in baseline, incorporating 10% of total myeloid cells at baseline (Fig. 2D and E; Fig. S2A). *FOSB* is a part of the AP-1 transcription factor complex that plays a key role in cytokine storms triggered by viruses like SARS (11, 18, 19). On the other hand, *NFKBIZ* regulates genes primarily involved in immune and inflammatory responses. The last AM-like cluster, mac_CHIT^hi^ (mac_7), highly expressed *CHIT1*, Cystatin B (*CSTB*), and Coiled-Coil-Helix-Coiled-Coil-Helix Domain Containing (*CHCHD*) *6* (Fig. 2D). *CHIT1* is a well-preserved and regulated chitinase secreted by activated macrophages. It is the primary chitinase in humans, capable of breaking down chitin and playing a significant role in the immune response, and it is involved in lung disease processes such as the pathogenesis of pulmonary fibrosis, bronchial asthma, chronic obstructive pulmonary disease, and pulmonary infections (20).

Besides macrophage clusters, our work identified one inflammatory DC cluster (DC), which expressed known DC marker FMS-like tyrosine kinase 3 (*FLT3*), also highly expressed Baculoviral IAP Repeat Containing 3 (*BIRC3*), C-C Motif Chemokine Ligands *CCL17*, *CCL22*, *MARCKS*, and *TMEM176B* (Fig. 2B and D). *BIRC3* was expressed on inflammatory monocyte-derived DCs and has the potential to be used as biomarkers for assessing matured DCs (21). Interactions between chemokine ligands and receptors coordinate leukocyte trafficking, impacting various critical cellular functions, such as the presentation of antigens and the production of cytokines by dendritic cells (22). The percentage of cells of this cluster was low at the baseline and then increased at 3 dpi, incorporating 0.62% (baseline) and 2.66% (3 dpi) of total myeloid cells in the respective time point in the BALs of macaques (Fig. 2E; Fig. S2A).

We identified two pDC populations, pDC_1 and pDC_2, which were significantly more frequent in the BAL of SARS-CoV-2 infected macaques at 3 dpi compared to earlier time points in the BAL of the infected macaques. These pDC populations were predominantly present at 3 dpi, constituted 0.32% at baseline to 20% at 3 dpi of total myeloid cells in the respective time point in the BALs of macaques (Fig. 2E; Fig. S2A). Both clusters expressed classic pDC markers such as Interleukin 3 Receptor subunit alpha (*IL3RA*), Leukocyte Immunoglobulin-like Receptor A4 (*LILRA4*), C-type lectin domain family 4 member C (*CLEC4C*), and Transcription Factor 4 (*TCF4*; Fig. 2D) (13, 15). These clusters also highly expressed Coiled-Coil Domain Containing 50 (*CCDC50*), Joining Chain of Multimeric IgA and IgM (*JCHAIN*), Interferon Regulatory Factors *IRF7*, *IRF8*, Selectin L (*SELL*), Derlin 3 (*DERL3*), Pre-T Cell Antigen Receptor Alpha (*PTCRA*), *SLC15A4*, *TLR7*, *TLR9*, *PACSIN1*, and *LAG3* (Fig. 2D; Fig. S2C). In addition, pDC_1 enriched IRF1 (Fig. S2C) and pDC_2 enriched Refilin B (*RFLNB*; Fig. 2D). *IRF7* and *IRF8* are important transcription factors for the induction of IFN responses. Known as the “master regulator” of Type I interferon-dependent immune responses, *IRF7* can be activated by viral detection pathways (23). Conversely, *IRF8* plays a distinct role in the maturation and specialization of myeloid cells, including macrophages and dendritic cells (24). *PACSIN1* is involved in the intracellular trafficking of receptors and has been implicated in immune responses by modulating the transport and signaling of Toll-like receptors and other receptors (25). The adhesion molecule, *SELL*, facilitates the migration of pDCs to high endothelial venules. *DERL3* and *LAG3* help maintain homeostasis, with *DERL3* ensuring protein quality control and *LAG3* involved in preventing overactive immune responses (26, 27). *SLC15A4* is known for its role in the activation of nucleic acid-sensing pathways in immune cells like *TLR7* and *TLR9*, ultimately contributing to the production of type I interferons (28). The intracellular receptors, *TLR7* and *TLR9*, specifically recognize viral RNAs and initiate signaling cascades that result in the production of type I interferons and pro-inflammatory cytokines (29). These genes work together to ensure effective immune surveillance, pathogen recognition, signaling to produce cytokines (especially type I IFNs), and the initiation of adaptive immune responses. They enable pDCs to fulfill their role as a critical bridge between innate and adaptive immunity.

We also identified two cDC populations: cDC1 and cDC2. The cDC1 cluster was highly enriched in *FLT3*, C-Type Lectin Domain Family 9 Member A (*CLEC9A*), *TCF4, IRF8*, Carboxypeptidase Vitellogenic Like (*CPVL*), Serpin Family B Member 9 (*SERPINB9*), and SLAM Family Member 7 (*SLAMF7*; Fig. 2B and D). On the other hand, the cDC2 cluster enriched *FLT3*, *TCF4*, *CD1A*, *CD1C*, and *WNT5A* (Fig. 2B and D). Both cDC1 and cDC2 clusters were also highly enriched in *MAMU-DQA1*, *MAMU-DQB1*, *MAMU-DRB1*, and *MAMU-DRA* (Fig. 2D), which encode Major Histocompatibility Complex (MHC) class II molecules, suggesting these clusters’ role as primary antigen-presenting cells in the immune system, bridging innate and adaptive immunity.

Therefore, our data represent the accumulation of multiple myeloid cells in the BAL following infection. In addition to cell type classification, we further explored the responses of these cells upon infection as discussed below.

### Myeloid populations respond rapidly by upregulating IFN signaling during SARS-CoV-2 infection

From our previous study, 3 dpi is the time of peak viremia following infection in our model, and rigorous IFN responses are expressed at this time (11). To understand the gene expression profiles that change in very early response to infection, we performed differential gene expression analysis followed by Reactome pathway analysis to compare 1 dpi/2 dpi/3 dpi vs baseline, separately, for each myeloid cluster.

**Fig 3:**
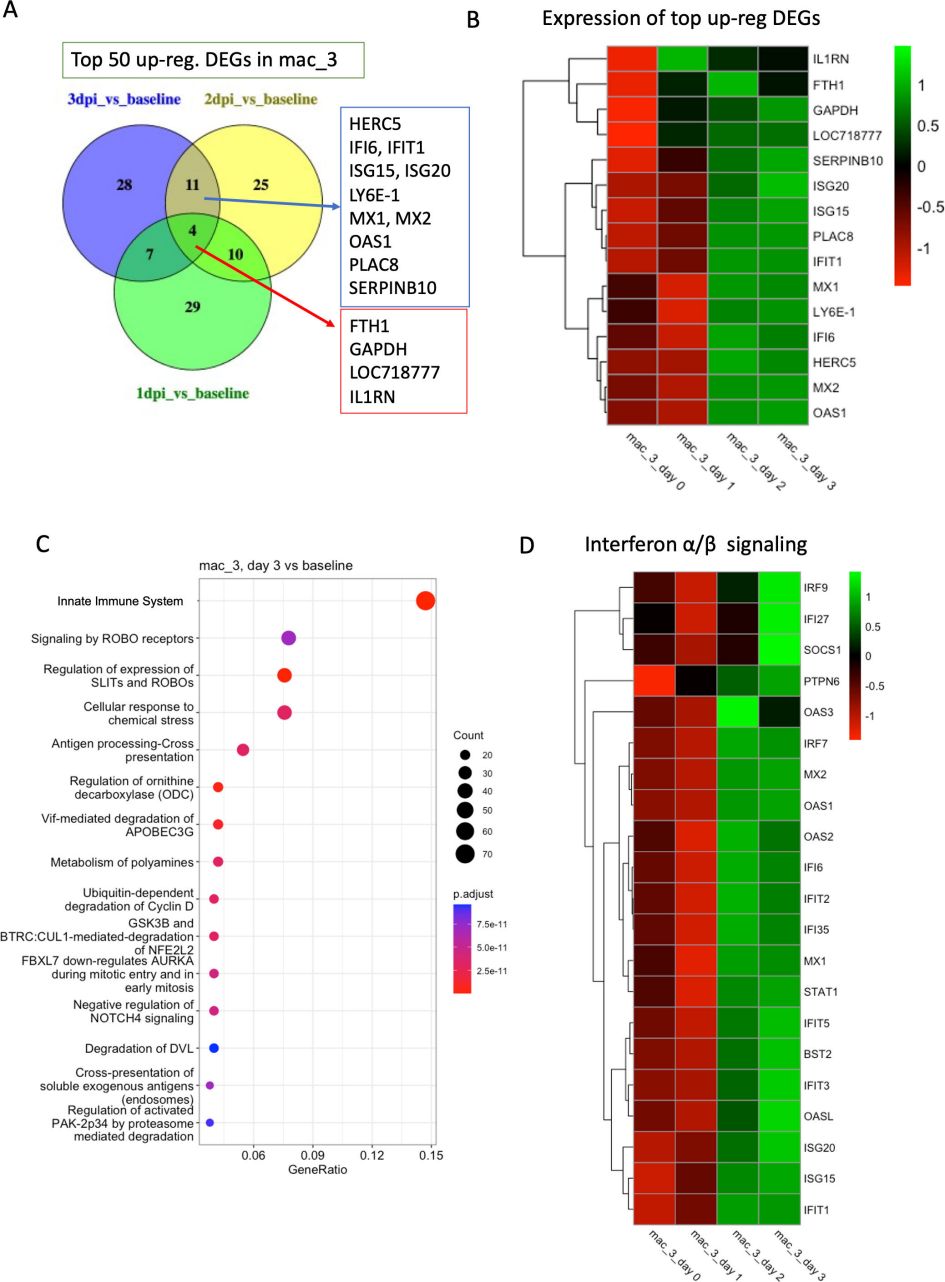
Transcriptomic analysis of mac_3. (**A**) Venn diagrams showing the common DEGs among the top 50 upregulated genes in different conditions (1 dpi/2 dpi/3 dpi) compared to baseline in mac_3. (**B**) Heatmap with average normalized expression of the top upregulated DEGs (listed in Fig. 3A) in different conditions (baseline, 1 dpi, 2 dpi, and 3 dpi). (**C**) Top 15 reactome pathways enriched at 3 dpi compared to baseline. (**D**) Heatmap with average normalized expression of the genes related to IFN-α/β signaling pathway in different conditions (baseline, 1 dpi, 2 dpi, and 3 dpi) in cluster mac_3.

Since the most cellular accumulation occurred at 3 dpi, we initiated analysis of clusters mac_3 and mac_5. In mac_3, we found 373 differentially expressed genes (DEGs) in 1 dpi vs baseline, 791 DEGs in 2 dpi vs baseline*,* and 1,295 DEGs in 3 dpi vs baseline. Several of these DEGs are associated with antiviral immune responses, often induced by type I IFN signaling. HECT and RLD Domain Containing E3 Ubiquitin Protein Ligase 5 (*HERC5*), *IFI6*, Interferon-Induced Protein With Tetratricopeptide Repeats 1 (*IFIT1*), Interferon-Stimulated Genes *ISG15*, *ISG20*, *LY6E-1*, MX Dynamin Like GTPase 1 and 2 (*MX1* and *MX2*), 2′-5′-Oligoadenylate Synthetase 1 (*OAS1*), and Placenta Associated 8 (*PLAC8*) were among the top 50 upregulated transcripts in both 3 dpi vs baseline and 2 dpi vs baseline but not 1 dpi vs baseline (Fig. 3A and B). *HERC5* catalyzes the ISGylation of target proteins, a process akin to ubiquitination that modifies proteins to help in the immune response against viral infections (30). *IFI6* inhibits viral replication and promotes cell survival. IFNs serve as the initial line of defense against viral infections and confine viral spread within the local area (31). *LY6E* is involved in the regulation of T cell proliferation, differentiation, and activation and has been shown to have a role in inhibiting the fusion of the SARS-CoV-2 Spike protein to the host cell membrane, thus acting as a defense mechanism against the virus (32–34). *MX1* and *MX2* can block viral replication by targeting viral nucleocapsids or nuclear import of viral components (35). *PLAC8* is involved in cell proliferation and apoptosis (36).

Pathway analysis identified the *innate immune system*, *cellular response to chemicals*, and *antigen processing-cross presentation* as among the top enriched pathways in 3 dpi compared to baseline in cluster mac_3 (Fig. 3C). The *innate immune system* is the first line of defense against any infection. The *cellular response to chemicals* includes various cellular reactions to internal and external chemical signals, such as cytokines, growth factors, and toxic substances. The genes involved in this pathway include 18 of the proteome subunits such as proteasome 20S subunit alpha (*PSMA2*, *PSMA6*, and *PSMA7*), proteasome 20S subunit beta (*PSMB1*, *PSMB3-7*, and *PSMB9-10*), proteasome 26S subunit, ATPase *PSMC2*, *PSMC5*, proteasome 26S subunit ubiquitin receptor, non-ATPase 2 *PSMD4*, *PSMD8*, *PSMD11*, *PSMD13*, and proteasome inhibitor subunit 1 *PSMF1*. Many of these are related to neurological disorders. In COVID-19, a key aspect of the cellular response to chemicals is the production and release of cytokines and chemokines by immune cells in response to the virus. These signaling molecules orchestrate the immune response by recruiting more immune cells to the site of infection, inducing inflammation, and promoting the clearance of the virus. Antigen processing-cross presentation is known to be crucial for the immune system to present viral antigens on the surface of certain immune cells and for the development of long-term immunity. This pathway includes *S100A8* and *S100A9*, along with the proteasome gene family. Studies have suggested that *S100A8/A9* has a regulatory effect on the cytokine storm, which is a defining feature of severe and often fatal COVID-19 cases (37). In addition to these pathways, IFN-α/β (Fig. 3D) and IFN-γ pathways were also enriched at 2 dpi and 3 dpi compared to baseline.

**Fig 4:**
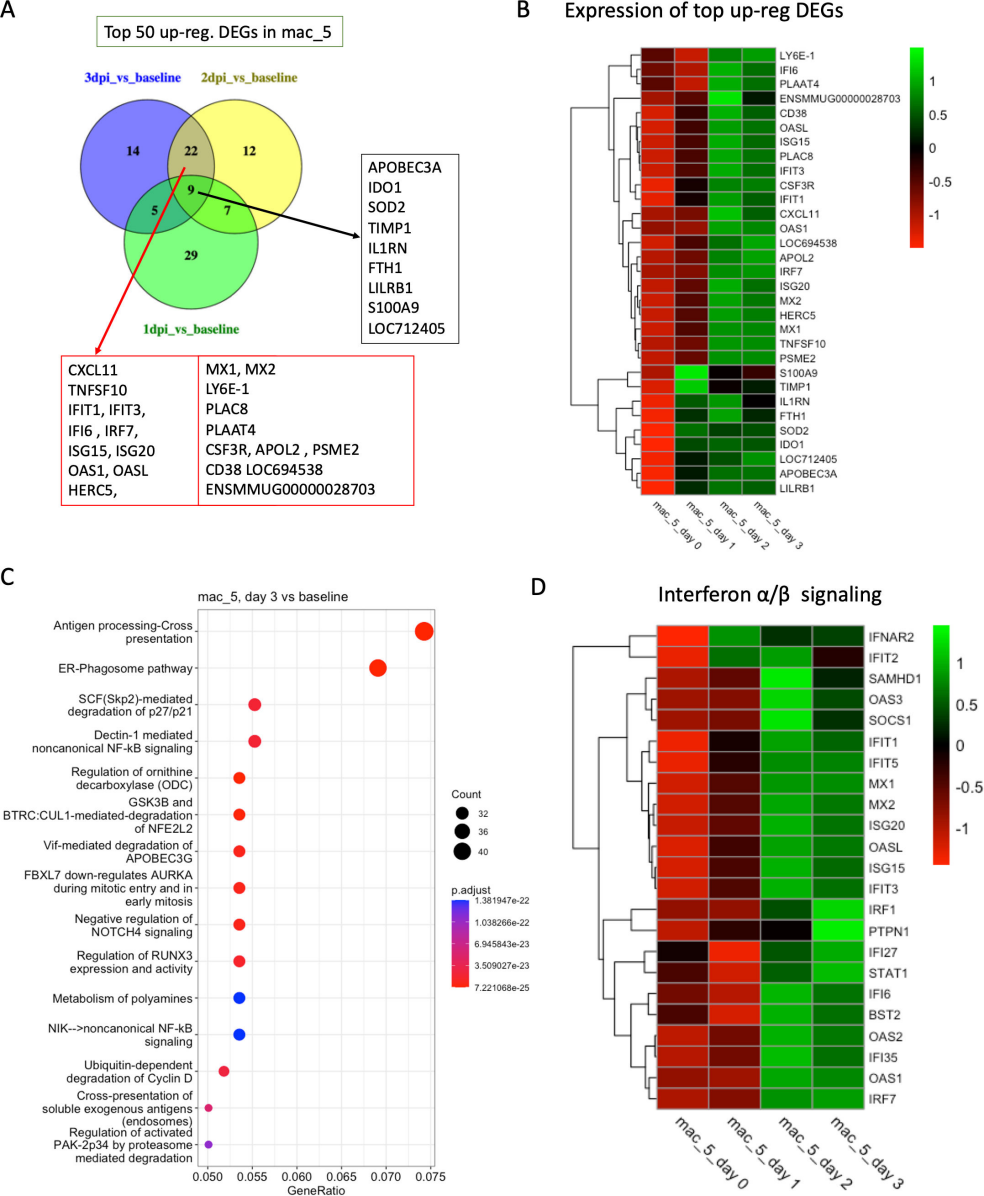
Transcriptomic analysis of mac_5. (**A**) Venn diagrams showing the common DEGs among the top 50 upregulated genes in different conditions (1 dpi/2 dpi/3 dpi) compared to baseline in mac_5. (**B**) Heatmap with average normalized expression of the top upregulated DEGs (listed in Fig. 4A) in different conditions (baseline, 1 dpi, 2 dpi, and 3 dpi). (**C**) Top 15 reactome pathways enriched at 3 dpi compared to baseline. (**D**) Heatmap with average normalized expression of the genes related to IFN-α/β signaling pathway in different conditions (baseline, 1 dpi, 2 dpi, and 3 dpi) in cluster mac_5.

In mac_5, we found 1,330 DEGs in 1 dpi vs baseline, 1,251 in 2 dpi vs baseline, and 1,876 in 3 dpi vs baseline in the infected BAL samples. The genes Apolipoprotein B mRNA Editing Enzyme Catalytic Subunit 3A (*APOBEC3A*), Indoleamine 2,3-Dioxygenase 1 (*IDO1*), Superoxide Dismutase 2 (*SOD2*), Tissue Inhibitor of Metalloproteinases 1 (*TIMP1*), Interleukin 1 Receptor Antagonist (*IL1RN*), Ferritin Heavy Chain 1 (*FTH1*), Leukocyte Immunoglobulin Like Receptor B1 (*LILRB1*), and S100A9 are among the top 50 upregulated differentially expressed genes in the infected BAL samples compared to baseline (Fig. 4A and B). Although these genes are involved in diverse processes, they are commonly implicated in regulating immune responses and inflammation. In addition, we found several genes upregulated in both 3 dpi vs baseline and 2 dpi vs baseline only which regulate immune responses and inflammation, such as C-X-C Motif Chemokine Ligand 11 (*CXCL11*), TNF Superfamily Member 10 (*TNFSF10*), *IFIT1*, *IFIT3*, *IFI6*, *IRF7*, *ISG15*, *ISG20*, *OAS1*, *OASL*, *HERC5*, *MX1*, *MX2*, *LY6E-1*, *PLAC8*, Phospholipase A and Acyltransferase 4 (*PLAAT4*), Colony Stimulating Factor 3 Receptor (*CSF3R*), *APOL2*, and Proteasome Activator Subunit 2 (*PSME2*; Fig. 4A and B). *APOBEC3A* has antiviral properties, particularly by introducing mutations into viral genomes (38). *SOD2* protects cells from damage by reactive oxygen species, which can be produced during immune responses to pathogens. *IL1RN* acts to limit the inflammatory response by inhibiting the activity of IL-1. *FTH1* can reduce its availability to pathogens and limit oxidative stress. *IFIT3* works with *IFIT1* to bind to viral RNA, preventing its translation and replication (39), whereas *IFI6* helps cells resist virus-induced apoptosis, and IRF7 regulates the expression of ISGs. *ISGs*, *OAS1*, and *OASL* enhance the antiviral response (40). Overall, these genes and proteins contribute to a robust antiviral defense, triggering various immune mechanisms designed to detect and destroy viral pathogens, regulate immune signaling and inflammation, and modulate cell death to contain infections.

Pathway analysis also identified *antigen processing-cross presentation*, *endoplasmic reticulum (ER)-phagosome pathway*, and *Dectin-1 mediated non-canonical NF-*κ*B signaling* pathway as the top enriched pathways at 3 dpi (Fig. 4C; Fig. S3A). The ER-phagosome pathway involves the fusion of the ER with phagosomes to form an ER-phagosome fusion compartment. This is known to play a role in enhancing the presentation of antigens derived from pathogens that are ingested by phagocytosis. Dectin-1 is a pattern recognition receptor that primarily recognizes β-glucans in fungal cell walls, but it can also modulate immune responses to other pathogens. The *non-canonical NF-κB signaling pathway*, which can be activated by Dectin-1, is involved in inflammatory responses and is slower acting than the canonical NF-κB pathway. It can contribute to the production of certain cytokines and the regulation of immune responses. Together, these pathways contribute to how the immune system detects, responds to, and attempts to eliminate SARS-CoV-2. In addition to these pathways, we also identified the *IL-1 signaling pathway* and the *IFN-α/β signaling* pathway, which are enriched at 3 dpi, and the expression of the genes involved in these pathways changes over the infection at different time points (Fig. 4D; Fig. S3B).

**Fig 5:**
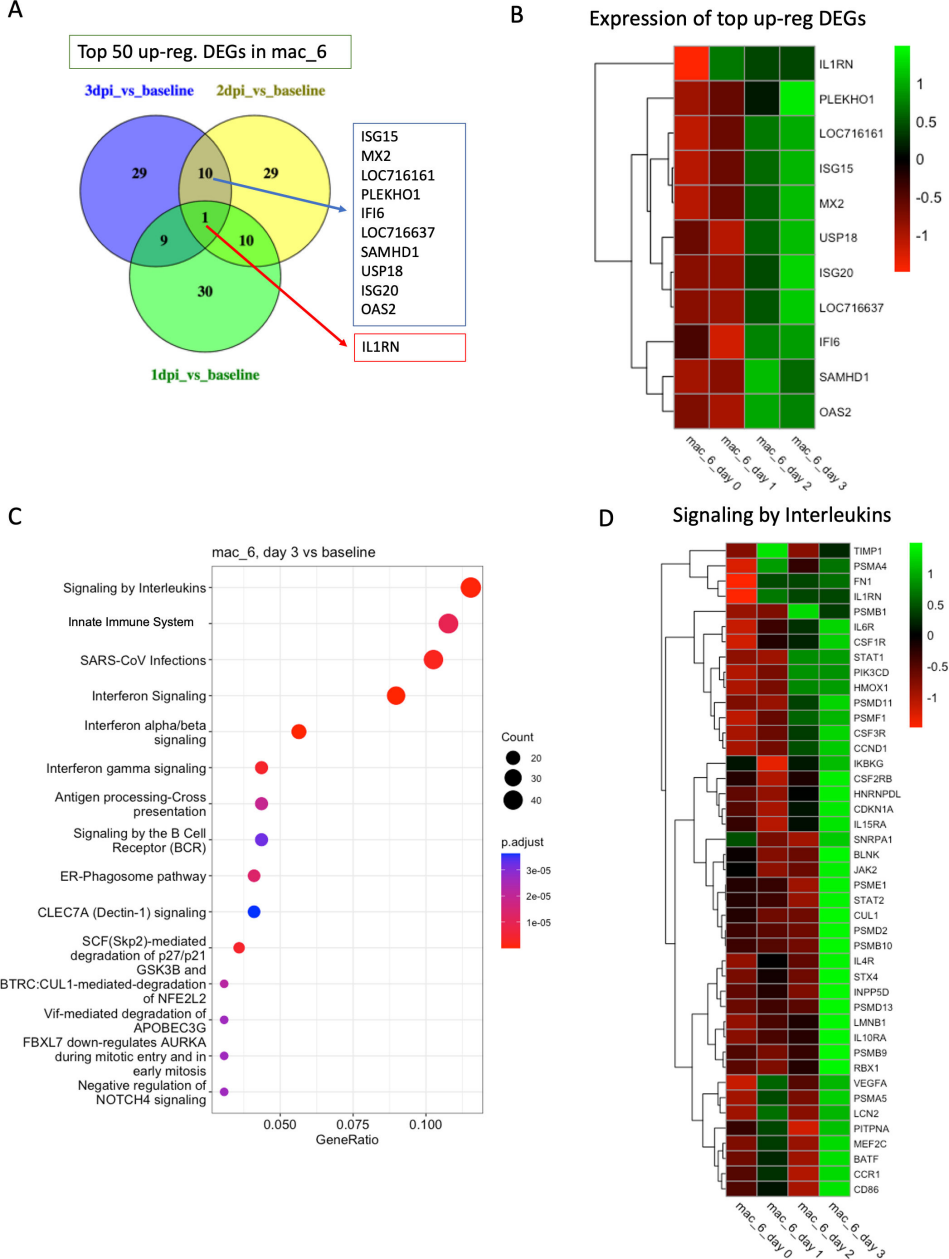
Transcriptomic analysis of mac_6. (**A**) Venn diagrams showing the common DEGs among the top 50 upregulated genes in different conditions (1 dpi/2 dpi/3 dpi) compared to baseline in mac_6. (**B**) Heatmap with average normalized expression of the top upregulated DEGs (listed in Fig. 5A) in different conditions (baseline, 1 dpi, 2 dpi, and 3 dpi). (**C**) Top 15 reactome pathways enriched at 3 dpi compared to baseline. (**D**) Heatmap with average normalized expression of the genes related to signaling by interleukins pathway in different conditions (baseline, 1 dpi, 2 dpi, and 3 dpi) in cluster mac_6.

In mac_6, we found 309 DEGs in 1 dpi vs baseline, 146 DEGs in 2 dpi vs baseline*,* and 1,159 DEGs in 3 dpi vs baseline. The genes *ISG15*, *ISG20*, *MX2*, *OAS2*, *IFI6*, *SAMHD1*, *USP18*, and *PLEKHO1* were upregulated in both 3 dpi vs baseline and 2 dpi vs baseline (Fig. 5A and B) and are commonly upregulated by interferon signaling. Enriched pathways in this cluster include *signaling by interleukins*, *innate immune system*, *SARS-CoV infections*, *IFN-α/β signaling*, *IFN-γ signaling*, and *antigen processing-cross representation* (Fig. 5C and D; Fig. S4A through C).

**Fig 6:**
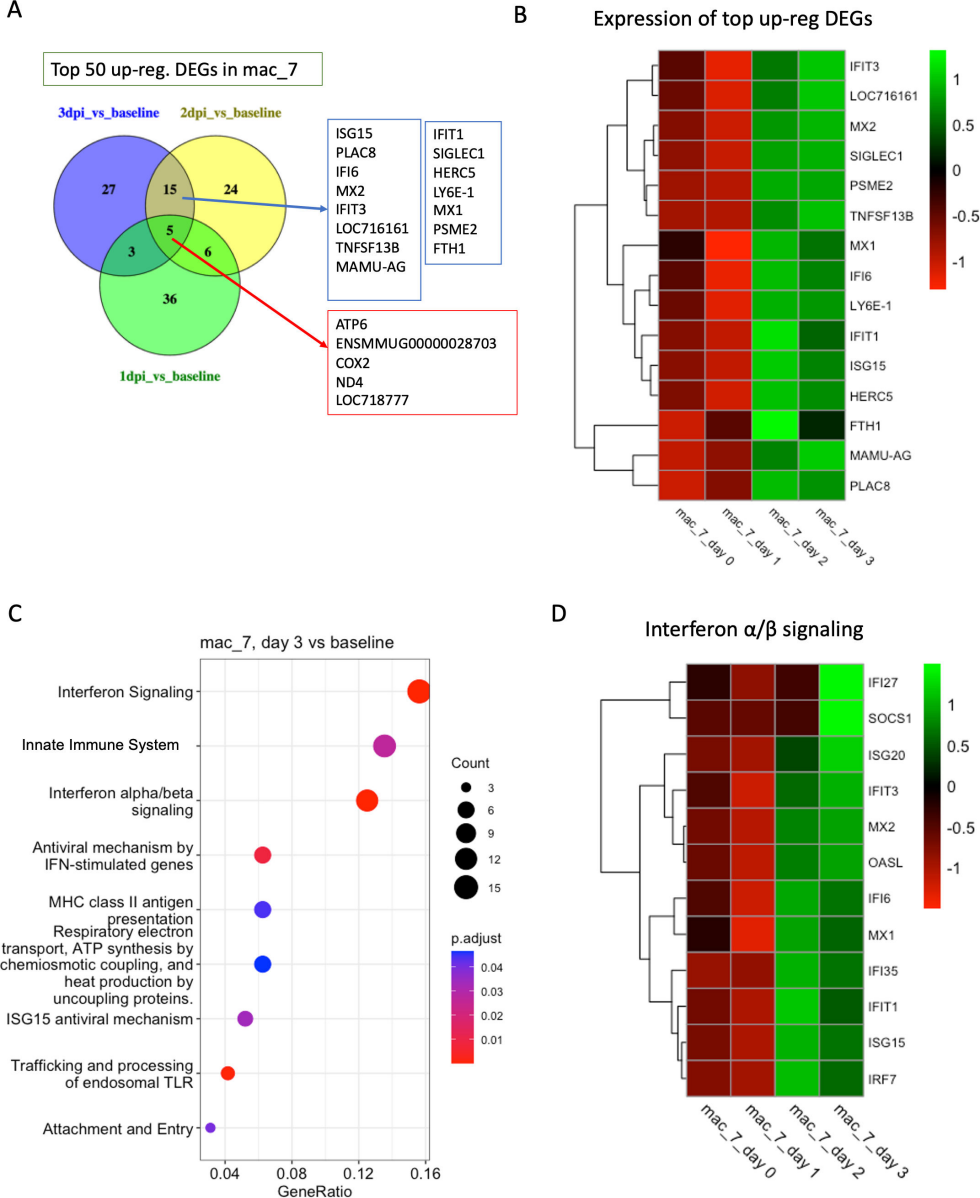
Transcriptomic analysis of mac_7. (**A**) Venn diagrams showing the common DEGs among the top 50 upregulated genes in different conditions (1 dpi/2 dpi/3 dpi) compared to baseline in mac_7. (**B**) Heatmap with average normalized expression of the top upregulated DEGs (listed in Fig. 6A) in different conditions (baseline, 1 dpi, 2 dpi, and 3 dpi). (**C**) Top 15 reactome pathways enriched at 3 dpi compared to baseline. (**D**) Heatmap with average normalized expression of the genes related to IFN-α/β signaling pathway in different conditions (baseline, 1 dpi, 2 dpi, and 3 dpi) in cluster mac_7.

On the other hand, in mac_7, we found 295 DEGs in 1 dpi vs baseline, 231 DEGs in 2 dpi vs baseline, and 342 DEGs in 3 dpi vs baseline. Interestingly, the genes *FTH1*, *HERC5*, *IFI6*, *IFIT1*, *IFIT3*, *ISG15*, *LY6E-1*, *MAMU-AG*, *MX1*, *MX2*, *PLAC8*, *PSME2*, Sialic Acid Binding Ig Like Lectin 1 (*SIGLEC1*), and Tumor Necrosis Factor Superfamily Member 13B (*TNFSF13B*) were upregulated in both 3 dpi vs baseline and 2 dpi vs baseline but not in 1 dpi vs baseline (Fig. 6A and B). These genes contribute to the regulation of iron metabolism (*FTH1*), modification of proteins in response to infection (*HERC5*), presentation of antigens to the immune system (*MAMU-AG*), control of apoptotic pathways (*IFI6* and *PLAC8*), and direct antiviral activities.

Enriched pathways in mac_7 include *IFN-α/β signaling*, *antiviral mechanism by IFN-stimulated genes*, *innate immune system*, *ISG15 antiviral mechanism*, *MHC class II antigen presentation*, and *respiratory electron transport*, *ATP synthesis by chemiosmotic coupling*, and *heat production by uncoupling proteins* (Fig. 6C and D). Collectively, these pathways create a multifaceted defense against COVID-19, involving both immediate antiviral actions and the regulation of the immune response to clear the virus and minimize damage to the host organism.

**Fig 7:**
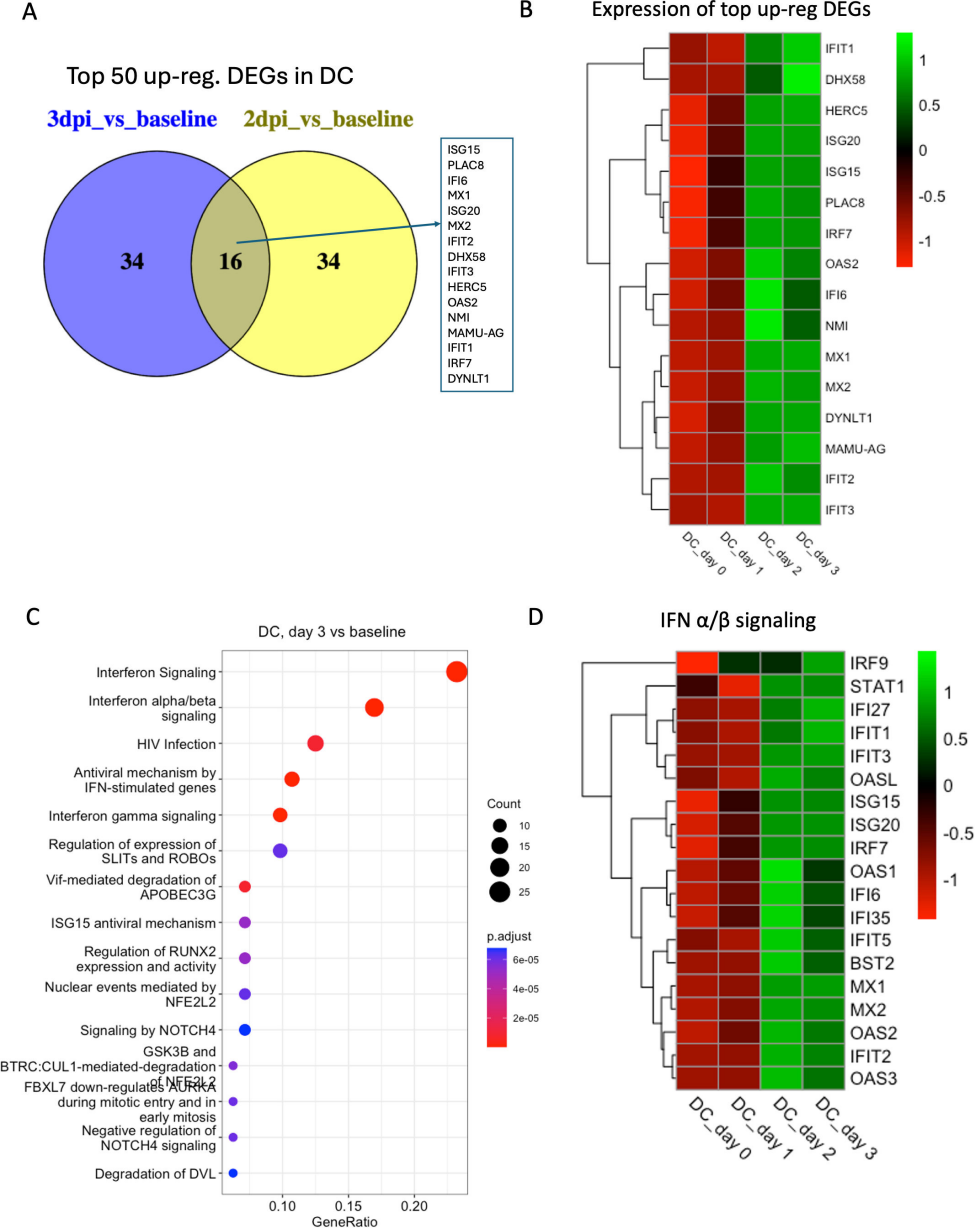
Transcriptomic analysis of cluster DC. (**A**) Venn diagrams showing the common DEGs among the top 50 upregulated genes in different conditions (1 dpi/2 dpi/3 dpi) compared to baseline in DC. (**B**) Heatmap with average normalized expression of the top upregulated DEGs (listed in Fig. 7A) in different conditions (baseline, 1 dpi, 2 dpi, and 3 dpi). (**C**) Top 15 reactome pathways enriched at 3 dpi compared to baseline. (**D**) Heatmap with average normalized expression of the genes related to IFN-α/β signaling pathway in different conditions (baseline, 1 dpi, 2 dpi, and 3 dpi) in cluster DC.

In the inflammatory DC cluster, we found 78 DEGs in 2 dpi vs baseline and 259 DEGs in 3 dpi vs baseline. The top common upregulated genes in 3 dpi vs baseline and 2 dpi vs baseline include several IFN-responsive genes, such as *HERC5*, *IFI6*, *IFIT1*, *IFIT2*, *IFIT3*, *IRF7*, *ISG15*, *ISG20*, *MAMU-AG*, *MX1*, *MX2*, *OAS2*, and *PLAC8* (Fig. 7A and B). The top enriched pathways were *IFN-α/β signaling*, *interferon signaling*, *antiviral mechanism by IFN-stimulated genes*, *IFN-γ signaling*, and *ISG15 antiviral mechanism* (Fig. 7C and D).

**Fig 8:**
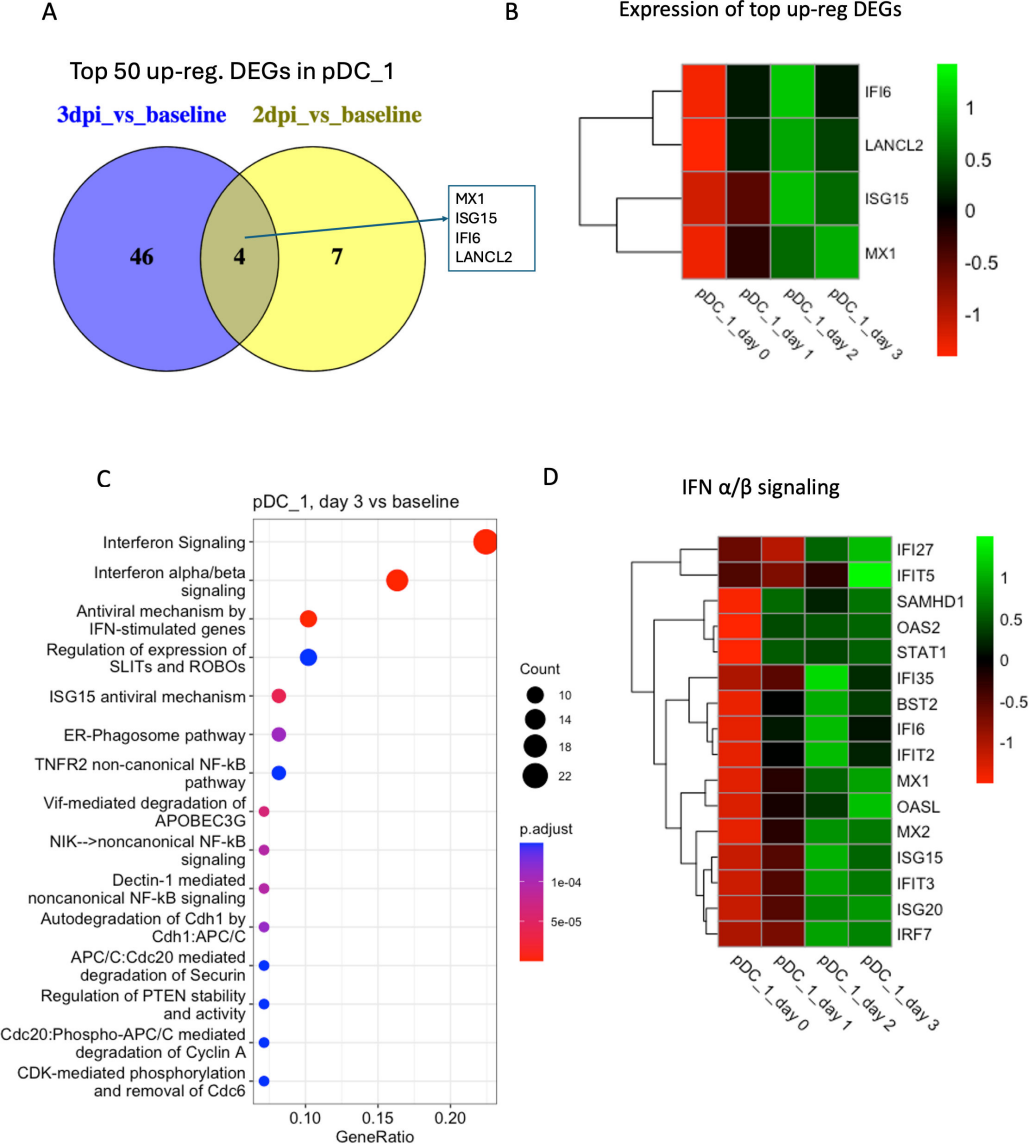
Transcriptomic analysis of cluster pDC_1. (**A**) Venn diagrams showing the common DEGs among the top 50 upregulated genes in different conditions (2 dpi/3 dpi) compared to baseline in pDC_1. No DEG was found in 1 dpi vs baseline. (**B**) Heatmap with average normalized expression of the top upregulated DEGs (listed in Fig. 8A) in different conditions (baseline, 1 dpi, 2 dpi, and 3 dpi). (**C**) Top 15 reactome pathways enriched at 3 dpi compared to baseline. (**D**) Heatmap with average normalized expression of the genes related to IFN-α/β signaling pathway in different conditions (baseline, 1 dpi, 2 dpi, and 3 dpi) in cluster pDC_1.

In the pDC_1 cluster, we found 11 DEGs in 2 dpi vs baseline and 237 DEGs in 3 dpi vs baseline. The top common upregulated genes in 3 dpi vs baseline include several IFN-responsive and MHC II genes: *IFI27, IFI6*, *IFIT2*, *IRF7*, *ISG15*, *ISG20*, *MAMU-A*, *MAMU-A3*, *MAMU-AG*, *MX1*, *MX2*, *STAT1*, and *TAP1* (Fig. 8A and B). The *IFN-α/β signaling*, *interferon signaling*, *antiviral mechanism by IFN-stimulated genes*, and *ISG15 antiviral mechanism* were among the top enriched pathways (Fig. 8C and D).

**Fig 9:**
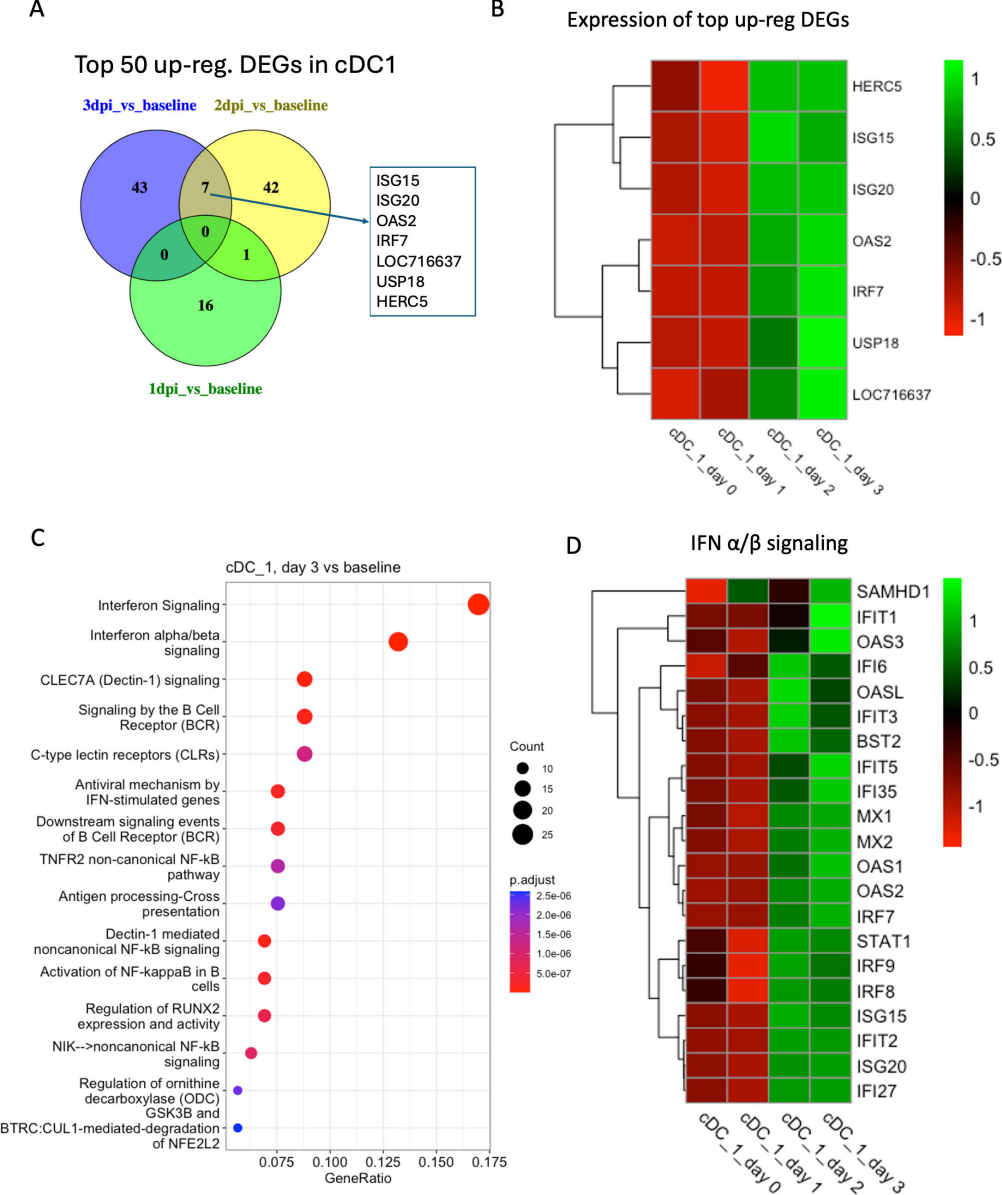
Transcriptomic analysis of cluster cDC1. (**A**) Venn diagrams showing the common DEGs among the top 50 upregulated genes in different conditions (1 dpi/2 dpi/3 dpi) compared to baseline in cDC1. (**B**) Heatmap with average normalized expression of the top upregulated DEGs (listed in Fig. 9A) in different conditions (baseline, 1 dpi, 2 dpi, and 3 dpi). (**C**) Top 15 reactome pathways enriched at 3 dpi compared to baseline. (**D**) Heatmap with average normalized expression of the genes related to IFN-α/β signaling pathway in different conditions (baseline, 1 dpi, 2 dpi, and 3 dpi) in cluster cDC1.

In the cDC1 cluster, 67 DEGs were upregulated in 2 dpi vs baseline and 281 DEGs in 3 dpi vs baseline. Several interferon-related genes were upregulated both at 2 dpi and 3 dpi, such as *ISG15*, *ISG20*, *OAS2*, *IRF7*, and *HERC5* (Fig. 9A and B). *IFN-α/β signaling*, *interferon signaling*, *CLEC7A (Dectin-1) signaling*, *Dectin-1-mediated non-canonical NF-kB signaling*, and *antiviral mechanism by IFN-stimulated genes* were the top enriched pathways at 3 dpi (Fig. 9C). In addition, *IFN-α/β signaling* was also enriched in this cluster at 2 dpi and 3 dpi compared to baseline (Fig. 9D).

**Fig 10:**
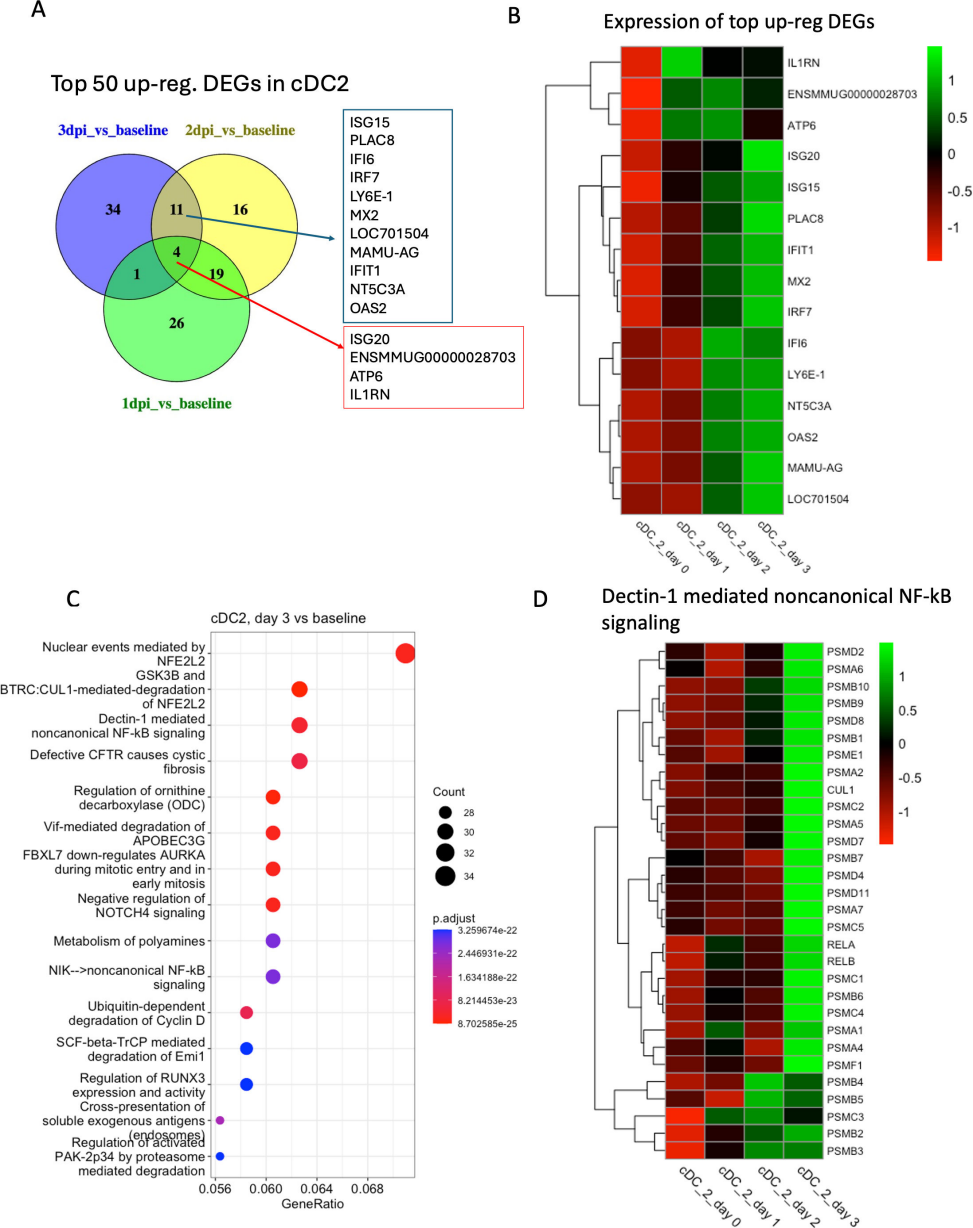
Transcriptomic analysis of cluster cDC2. (**A**) Venn diagrams showing the common DEGs among the top 50 upregulated genes in different conditions (1 dpi/2 dpi/3 dpi) compared to baseline in cDC2. (**B**) Heatmap with average normalized expression of the top upregulated DEGs (listed in Fig. 10A) in different conditions (baseline, 1 dpi, 2 dpi, and 3 dpi). (**C**) Top 15 reactome pathways enriched at 3 dpi compared to baseline. (**D**) Heatmap with average normalized expression of the genes related to Dectin-1-mediated non-canonical NF-κB signaling pathway in different conditions (baseline, 1 dpi, 2 dpi, and 3 dpi) in cluster cDC2.

In the cluster cDC2, as many as 502 DEGs were upregulated in 2 dpi vs baseline and 920 DEGs in 3 dpi vs baseline. The top regulated genes in cDC2 included the type I IFN-related *ISG15*, *OAS2*, *IFI6*, *IRF7*, *IFIT1*, *MX2*, *PLAC8*, and *Ly6E-1* (Fig. 10A and B). *Dectin-1-mediated non-canonical NF-kB signaling* and *negative regulation of NOTCH4 signaling* were the top enriched pathways at 3 dpi (Fig. 10C and D). We also found *IFN-α/β signaling* was enriched in this cluster at 2 dpi and 3 dpi compared to baseline (Fig. S5).

Thus, our results show that myeloid subpopulations exhibited changes over time in response to SARS-CoV-2 infection. Notably, we identified several key pathways that were consistently enriched across most myeloid clusters starting at 2 dpi to 3 dpi, as discussed below.

### IFN and IL-1 and non-canonical NFKB signaling pathways are enriched in myeloid populations by 3 dpi coinciding with peak viremia

**Fig 11:**
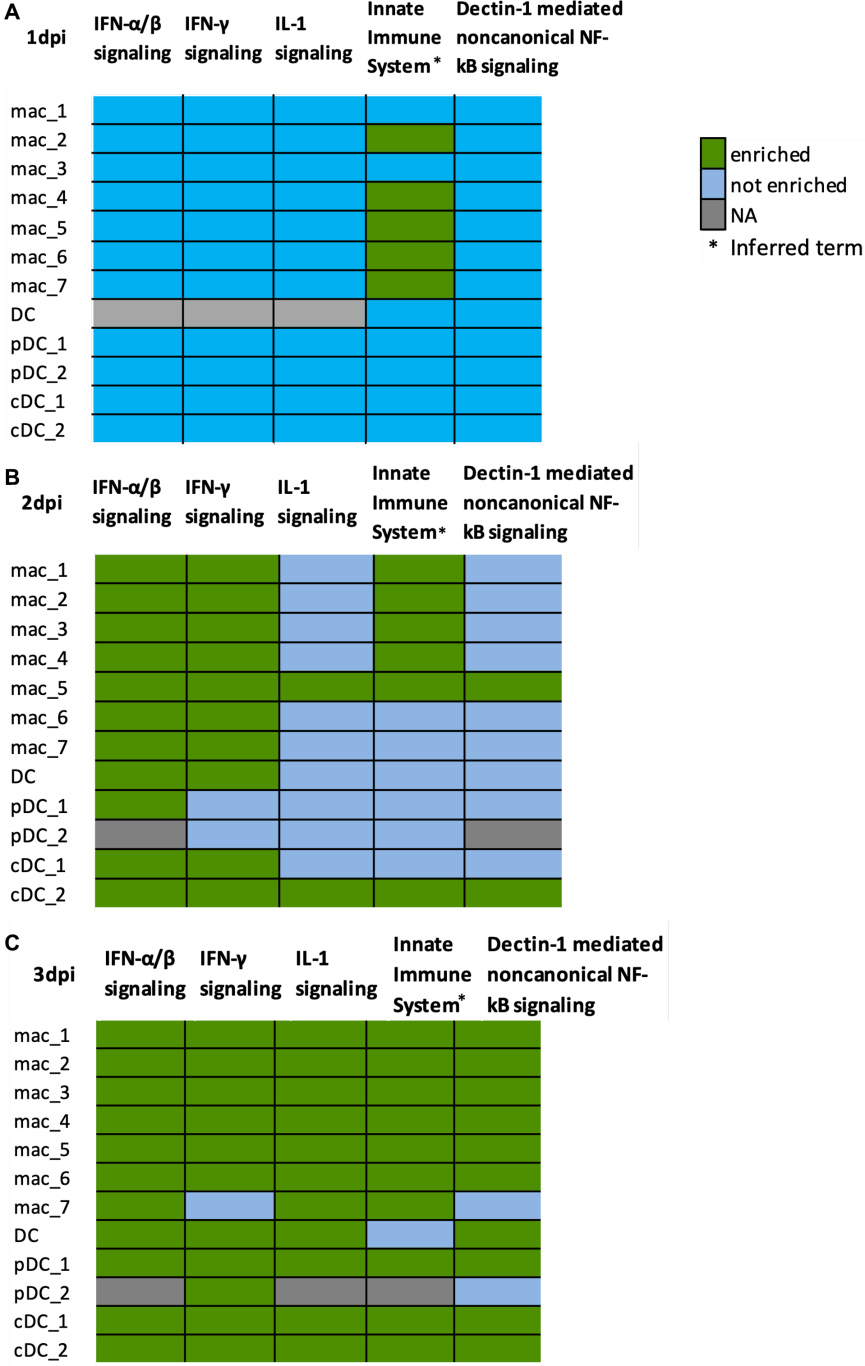
IFN, IL-1, and non-canonical NF-κB signaling pathways are enriched in myeloid cells by 3 dpi. Heatmap showing most common enriched reactome pathways in different myeloid populations at (**A**) 1 dpi, (**B**) infected at 2 dpi, and (**C**) infected at 3 dpi, all compared to baseline. Enriched (green), not enriched (blue) pathways, and NA (gray) for no pathway analysis performed due to lack of DEGs.

The pathway *innate immune system* was enriched as soon as 1 dpi compared to baseline in several macrophage populations, such as mac_2, mac_4, mac_5, mac_6, and mac_7 (Fig. 11A). By 2 dpi, we found *IFN-α/β signaling* was enriched in most clusters of myeloid populations (except pDC_2) and *IFN-γ signaling* in most of the myeloid populations (except pDCs), compared to baseline (Fig. 11B). Moreover, by 3 dpi, almost all clusters of myeloid populations were enriched by *IFN-α/β signaling*, *IFN-γ signaling*, *IL-1 signaling*, *innate immune system*, and *Dectin-1-mediated non-canonical NF-*κ*B signaling*, while compared to baseline (Fig. 11C).

IFN-α/β are cytokines that are quickly produced in response to viral infections and can inhibit viral replication. During SARS-CoV-2 infection, these cytokines aim to limit the spread of the virus by activating genes that interfere with the virus’s ability to replicate. The *IFN-γ signaling* helps in the activation of macrophages and the induction of the MHC class I and II molecules, enhancing antigen presentation to T cells, which is an important part of the immune response to viral infections such as SARS-CoV-2. The IL-1 family of cytokines plays a role in the inflammatory response. IL-1β is a potent pro-inflammatory cytokine, and the *IL-1 signaling* pathway can lead to fever and the production of other pro-inflammatory cytokines and chemokines, which can help in controlling SARS-CoV-2 by recruiting immune cells to the site of infection. The *innate immune system* is the body’s first line of defense against pathogens, including viruses like SARS-CoV-2. It includes physical barriers, such as the skin and mucous membranes, as well as immune cells like macrophages, dendritic cells, and natural killer cells. The *Dectin-1-mediated non-canonical NF-*κ*B signaling* pathway involves the activation of NF-κB, a protein complex that controls the transcription of DNA, cytokine production, and cell survival. It mediates activation of the p52/RelB NF-κB complex, thus regulating distinct immunological functions (41). The non-canonical pathway is slower and often involved in lymphoid organ development and adaptive immune response. These pathways work together to recognize and respond to pathogens non-specifically and quickly, often before the adaptive immune system is activated.

## DISCUSSION

A comprehensive understanding of the early host immune responses during SARS-CoV-2 infection is crucial to identify the factors that contribute to protection or pathology, especially in the first few days after the infection. This stage is pivotal because it signifies the onset of the host’s protective responses to combat the virus. Insights into the lung airways landscape at a very early time window following SARS-CoV-2 infection are not available to date. Moreover, there are several studies with human BAL scRNA-seq samples (4, 42, 43), but collecting human samples at the onset of infection is not feasible. In this study, we have applied scRNA-seq to understand the in-depth immune landscape of BAL at baseline and following SARS-CoV-2 infection up to the peak viremia at 3 dpi in the NHP model, a model which we recently developed to study COVID-19 (7).

The landscape analysis of the macaque’s lung immune microenvironment revealed dynamic changes in the proportions and characteristics of myeloid cells during infection. In particular, we identified eight distinct subpopulations of macrophage cells and five unique subpopulations of dendritic cells based on their unique gene expression patterns and their transcriptomic changes from baseline to 3 dpi. Our results showed that all macrophage cells in the BAL are AM-like populations, where two of the macrophage populations and all pDC populations are predominantly present at 3 dpi. Moreover, transcriptional analyses across different myeloid clusters identify several enriched pathways such as the *IFN signaling* pathway, *IL-1 signaling* pathway, and *Dectin-1-mediated non-canonical NF-*κ*B signaling* pathways.

Interestingly, we observed a shifting landscape among the DC subpopulations in NHP BAL samples during SARS-CoV-2 infection. Among the DC subsets, we observed an increase in the abundance of *BIRC3* and *FLT3*-expressing inflammatory DC population at 3 dpi. Of particular interest was the identification of pDC subsets (pDC_1 and pDC_2) that exhibit substantial enrichment at 3 dpi, in line with previous work that indicated the early involvement of pDCs during the initiation of viral infections (44). These pDC subsets were characterized by the classic pDC markers such as *IL3RA*, *LILRA4*, *CLEC4C*, and *TCF4* (45). These cells were also observed to induce high expression of the genes involved in the antiviral responses and pathogen-associated molecular patterns recognition, including *IRF7*, *IRF8*, *TLR7*, and *TLR9* (46), highlighting the key role of this population in initiating protective innate response against SARS-CoV-2 infection.

Additionally, conventional DC subpopulations (cDC1 and cDC2) displayed significant upregulation in the expression of the genes involved in antigen presentation and interferon responses, such as *CLEC9A*, *CD1C*, and *WNTA5A* in cDC1 and CD1A and CD1C in cDC2 (47). This indicates their pivotal role in bridging innate and adaptive immune responses. Thus, overall, our study highlighted the dynamic involvement of various DC subsets in generating a robust immune reaction through antigen presentation, interferon signaling, and activation of innate immune pathways during the early stages of SARS-CoV-2 infection in the NHP model.

Our study found that both IFN-α/β and IFN-γ transcripts are overexpressed in the BAL myeloid clusters in SARS-CoV-2-infected macaques compared to baseline. Increased IFN signature is consistent with the transcriptional signatures reported in other COVID studies and correlates with the severity of COVID-19 (4, 11, 48, 49) and suggests that IFN-responsive genes at 2 dpi (early stage of SARS-CoV-2 infection with peak viral titer in ferret model) could be advantageous in clearing SARS-CoV-2 (12). Both IFN-α/β and IFN-γ play roles in protecting the host, yet IFN-α/β may also lead to harmful outcomes depending on the immune environment encountered by tuberculosis (50–52).

IFNs are widely recognized for their role in combating viral infections, but they also play a crucial role in controlling cell proliferation and modulating the immune system. It is increasingly evident that these cytokines are key players in both innate and adaptive immune responses. IFN-α/β signaling is among the first cytokines produced by the host cells in response to viral infection (53). They bind to the IFN-α/β receptor on cells, triggering a signaling cascade that results in the expression of ISGs (53). These ISGs encode proteins with antiviral properties that help to inhibit viral replication and spread (54). The role of IFN-γ signaling is more related to the modulation of the immune system, enhancing the antimicrobial activities of macrophages and influencing the adaptive immune response. A key function of IFN-γ involves stimulating macrophages to boost their capacity for destroying pathogens, killing tumor cells, and eliminating intracellular microbes (55). Hence, our findings indicate that IFN-α/β and IFN-γ responses might have varying impacts during initial infections, necessitating further investigation.

IL-1 is a pro-inflammatory cytokine that can contribute to the inflammation observed in patients with COVID-19, particularly in severe cases (56). Some patients with severe COVID-19 develop a cytokine storm, which is an excessive and uncontrolled release of pro-inflammatory cytokines, including IL-1. This can lead to severe inflammation, tissue damage, and organ failure (57). Studies also suggest that the surge of pro-inflammatory cytokines is linked with an increased risk of severe pneumonia, ARDS, and failure of multiple organs (58). Indeed, it has been observed that the uncontrolled and upregulated pro-inflammatory cytokines have a direct relationship with the intensity and fatality rates of SARS-CoV-2 infections (59). The IL-1 contributes to the inflammatory cascade that can lead to the activation of both innate and adaptive immunity (60), helps recruit immune cells to the site of infection, and induces fever, which can enhance immune function (61). It promotes the production of secondary cytokines and chemokines, which further amplify the immune response (61). IL-1 is involved in the inflammatory response that can be triggered by SARS-CoV-2 infection (62).

In this study, we found the coexistence of high IFN and IL-1 in the macrophage, DCs, pDC, and cDC populations in the airways of SARS-CoV-2 infected macaques. The coexistence of high IFN and IL-1 levels in COVID-19 could reflect the virus’s ability to initially evade the immune response, potentially leading to a delayed and uncontrolled immune reaction. scRNA-seq on immunocytes in PMBCs from patients with healthy, mild, or severe COVID-19 and severe influenza found that while severe influenza patients primarily exhibited IFN responses, COVID-19 patients showed distinctive hyperinflammatory, TNF- and IL-1β-driven responses (49). Notably, in classical monocytes, type I IFN responses were present alongside TNF and IL-1β in patients with severe COVID-19 but not in mild COVID-19 cases (49). In addition, elevated levels of IFN-λ were found to be correlated with reduced viral load in bronchial aspirates and quicker viral clearance. Furthermore, a higher ratio of IFN-λ to type I IFN was associated with better outcomes in critically ill COVID-19 patients in a study of 32 COVID-19 patients and 16 flu patients (63). Understanding these dynamics helps in the development of targeted therapies and in managing the disease more effectively.

These findings provide in-depth insights into the immune cell dynamics in the lung airways of SARS-CoV-2 infected macaques, enhancing our fundamental knowledge of COVID-19 immunology. These also help to characterize animal models that are crucial for the evaluation of COVID-19 vaccines and therapeutics.

### Limitations of the study

Our study has some limitations. Our research specifically focused on the initial stages of infection within the BAL compartment, where myeloid cells were the predominant cell type. Therefore, we performed a comprehensive analysis exclusively of the myeloid cells. We collected BAL fluid from the rhesus macaques daily for 4 continuous days. Since immune cells do not get replenished every day, some samples, especially those from days 1 and 2 post-infection, had fewer cells. This highlights the inherent challenge of recovering viable immune cells during the early stages of infection, particularly from a site like the BAL, where cell numbers are naturally low. In addition, at early time points such as 1–2 dpi, we did not have the threshold or the samples to detect cellular responses reproducibly or to confirm by quantitative polymerase chain reaction. We also acknowledge that increasing the number of animals per time point would enhance the statistical power of the study. However, due to logistical as well as ethical considerations inherent in nonhuman primate research, we were limited in the number of animals used. However, in our previous study, we stringently assessed and correlated scRNA-seq readouts at later time points and confirmed similar findings by confocal and flow cytometry (11).

Despite these limitations, we ensured rigorous quality control and statistical methods, including aggregation of data across replicates and appropriate normalization techniques, to minimize potential biases. In addition, the cell counts were sufficient to perform differential analysis, which identified top markers in each cluster and significant transcriptomic differences within each cluster due to the infection.

## MATERIALS AND METHODS

### Experimental model and subject details

#### Macaques

This study did not involve any live rhesus macaques. Samples obtained from rhesus macaques (*Macaca mulatta*) of Indian origin infected with 1.05 × 10^6^ pfu SARS-CoV-2 isolate USA-WA1/2020 (BEI Resources, NR-52281, Manassas, VA) through ocular, intranasal, and intratracheal routes. The Texas Biomedical Research Institute’s Biohazard and Safety Committee and the Institutional Animal Care and Use Committee granted approval for all original experimental procedures which have been published earlier (7). The viral stock used for exposure was verified as SARS-CoV-2 through deep sequencing and matched the sequence previously published in GenBank (MN985325) for the strain USA-WA1/2020 (provided by BEI Resources, NR-52281).

#### Isolation of BAL single cells from macaques

Single-cell suspensions from BAL, collected at different time points as previously described (7, 64), were cryopreserved in Cryostor-CS10 (Biolife Solutions, USA) at −70°C. These samples were then used for downstream processing of scRNA-seq.

#### Single-cell RNA: library generation and sequencing

scRNA-seq was done following the manufacturer’s protocol (10× genomics) as previously described (13). The frozen BAL single-cell suspension was rapidly thawed in a water bath, and 2 × 10^6^ cells were allocated for downstream processing. These BAL single-cell suspensions were subjected to droplet-based massively parallel single-cell RNA sequencing in the BSL-3 laboratory using the Chromium Single Cell 3′ (v3.1) Reagent Kit, in accordance with the manufacturer’s instructions (10× Genomics). Briefly, cell suspensions were prepared at a concentration of 1,000 cells/µL, targeting the capture of 10,000 cells per lane. The 10× Chromium Controller then produced GEM droplets, within which each cell received a unique barcode, and during reverse transcription, each transcript was marked with a unique molecular identifier (65). The barcoded cDNA was extracted and taken out of the BSL-3 to generate libraries. This cDNA was amplified for 11 cycles and then subjected to fragmentation, end repair, A-tailing, adapter ligation, and sample index PCR according to the manufacturer’s guidelines. The libraries were then sequenced using a NovaSeq S4 (200 cycles) flow cell, aiming for 30,000 read pairs per cell.

#### Single-cell RNA-seq data processing

The raw gene expression matrices were generated by the Cell Ranger software (10× Genomics, version 3) available on the 10× website. After demultiplexing, the resulting fastq files were aligned against the *Macaca mulatta* (rhesus monkey) genome assembly Mmul_10 (rheMac10) with cellranger count. For each sample, the recovered-cell parameter was set to 10,000 cells that we expect to recover for each library. The output-filtered gene count matrices were analyzed by R software (4.3.1) with the Seurat (66) package (4.3.0.1). Different thresholds were chosen because of their distinct distribution in each sample to get good-quality cells (67). We filtered out cells that had more than 5% of mitochondrial genes. Samples with similar conditions were merged, data were normalized with default parameters, and most variable genes were detected by the *FindVariableFeatures* function. All samples were combined using Seurat functions, *FindIntegrationAnchors* and *IntegrateData. ScaleData* was used to regress out the number of unique molecular identifiers and mitochondrial content, and principal component analysis (PCA) was performed with *RunPCA*. The t-distributed stochastic neighbor embedding (tSNE) dimensionality reduction was performed on the scaled matrix using the first 30 PCA. For clustering, the *FindNeighbors* (20 PCA) and *FindCluster*s (resolution 0.5) functions were used. *FindAllMarkers* was used to compare a cluster against all other clusters to identify the marker genes. For each cluster, the minimum required average log fold change in gene expression was set to 0.25, and the minimum percent of cells that must express genes in either cluster was set to 25%. To re-analyze myeloid sub-populations, we pooled the clusters that we identified as myeloid origin and re-ran PCA, tSNE, and clustering to get a better resolution for further analysis. The function *FindMarkers* was used to compare the cells in a selected cluster across different conditions. The analysis was performed based on the Wilcoxon rank sum test with thresholds average logFC ≥ 0.25 and adj-*P*-value ≤ 0.05 using Seurat. Reactome pathway analysis was performed using ReactomePA (68). Several R packages were used to generate figures and intermediate data preprocessing, such as ggplot2 (69) and biomaRt (70).

## Data Availability

Single-cell RNA-seq data have been deposited at GEO. The GEO accession number for the raw and processed data for sc-RNAseq generated for this study is GSE293992. Any additional information required to reanalyze the data reported in this paper is available from the lead contact upon request.

## SUPPLEMENTAL MATERIAL

Supplementary figures spectrum.02715-24-s0001.docx Supplementary figures with legends.

ASM does not own the copyrights to Supplemental Material that may be linked to, or accessed through, an article. The authors have granted ASM a non-exclusive, world-wide license to publish the Supplemental Material files. Please contact the corresponding author directly for reuse.
