# Supplementary material for: Single-cell transcriptome analysis of bronchoalveolar lavage during early SARS-CoV-2 infection
Source: Microbiol Spectr. 2025 Jul 31;13(9):e02715-24. doi: 10.1128/spectrum.02715-24 (PMC12403745; doi:10.1128/spectrum.02715-24)
Supplement: Supplementary figures — with legends. [file spectrum.02715-24-s0001.docx]

**Supplementary Information**

**Single-cell transcriptome analysis of bronchoalveolar lavage during early SARS-CoV-2 infection**

Sadia Akter^1#^, Mushtaq Ahmed^1#^, Dhiraj K. Singh^2^, Kuldeep S. Chauhan^1^, Deepak Kaushal^2^, Shabaana A. Khader^1,3,*^

^1^Department of Microbiology, The University of Chicago, Chicago, IL 60637, USA.

^2^Southwest National Primate Research Center and Host Pathogen Interactions Program, Texas Biomedical Research Institute, San Antonio, TX 78227, USA.

^#^Equal authorship

^3^Lead Contact

^*^Correspondence: Khader@uchicago.edu

**
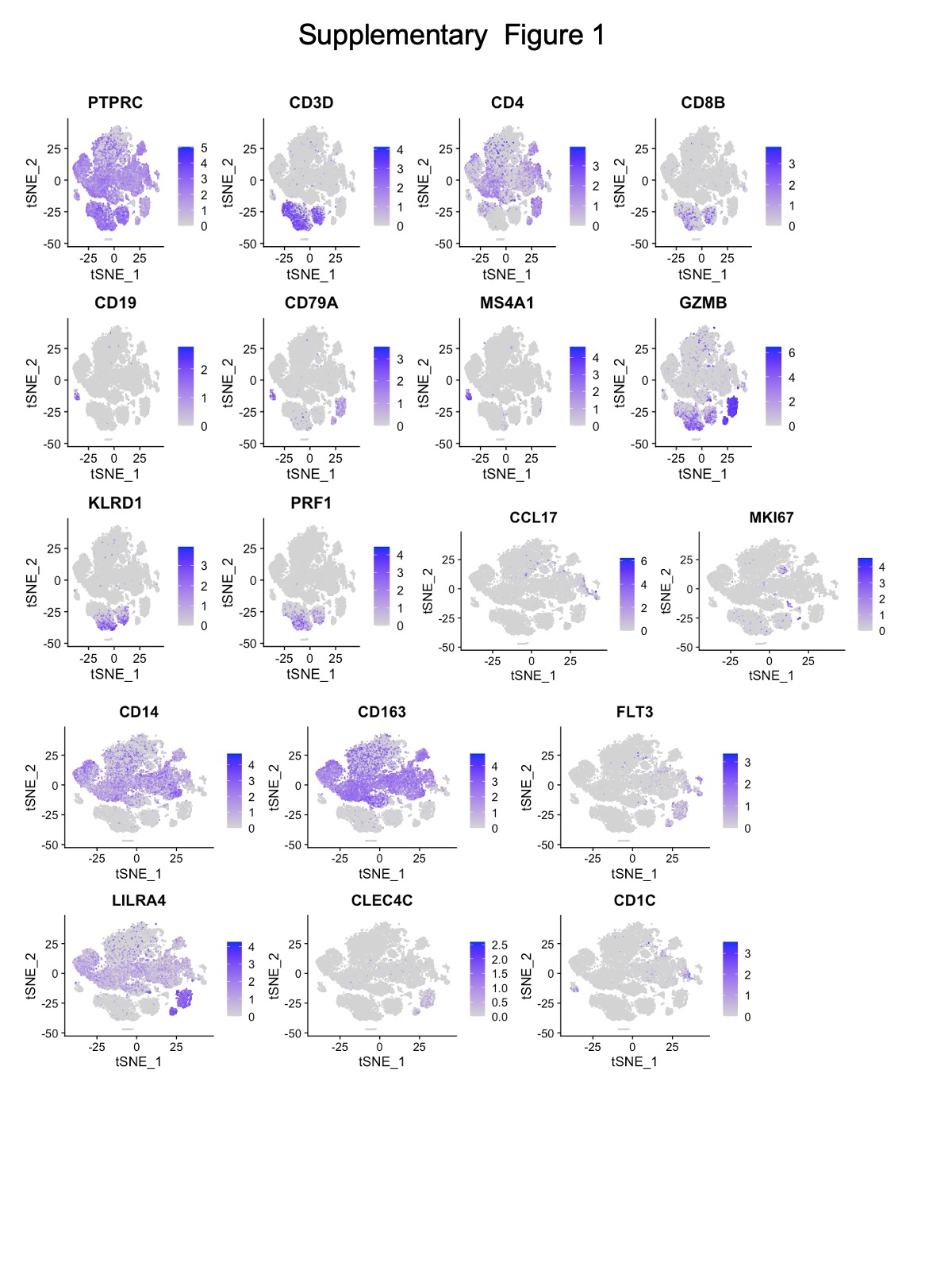
Suppl. Figure 1, related to Fig 1: tSNE plot with the expression of known cell markers.** Baseline (n=3), infected at 1dpi (n=4), infected at 2dpi (n=3), infected at 3dpi (n=4). The expressions of these marker genes were used to characterize distinct clusters according to their cell identity.

**
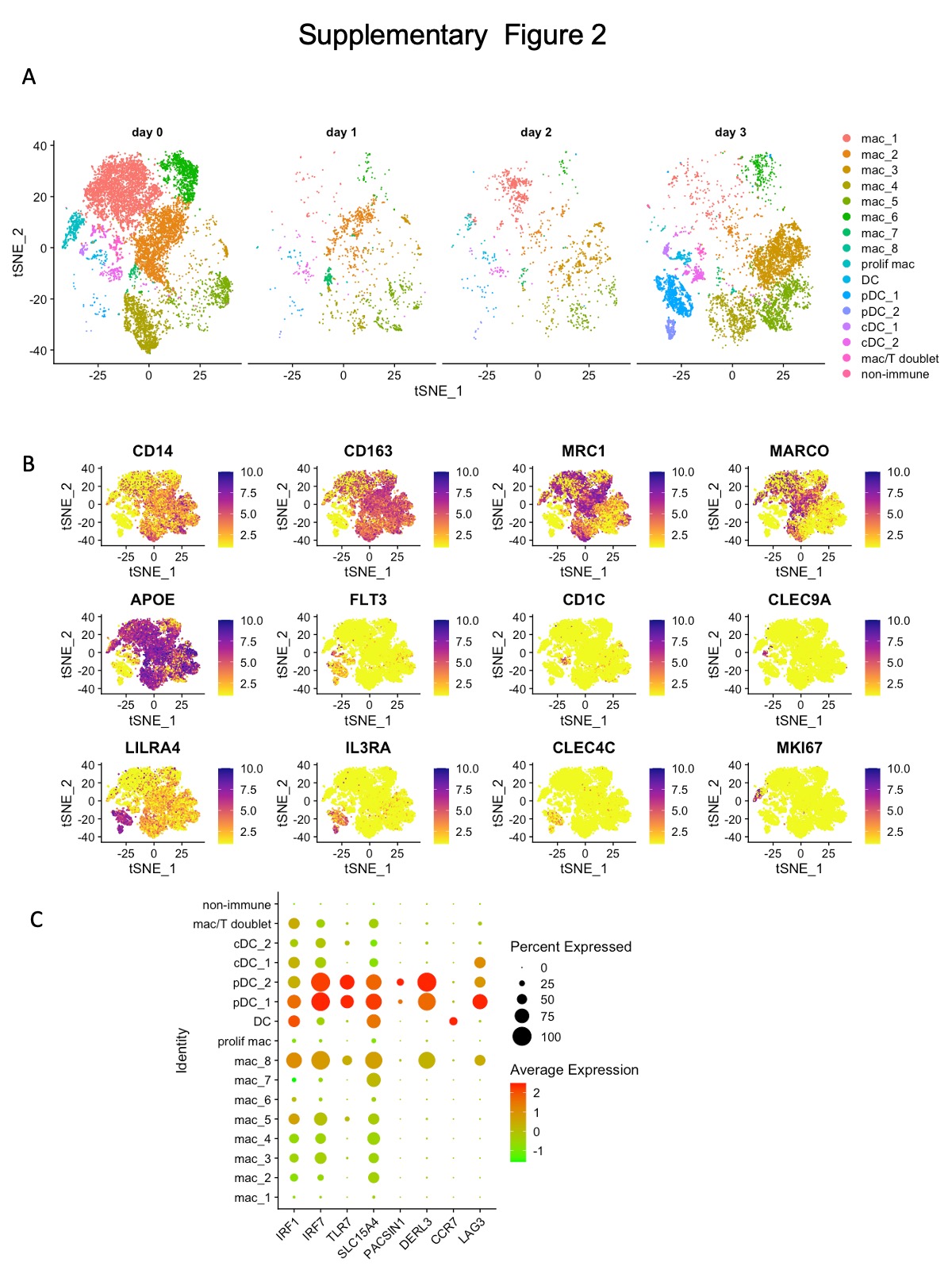
Suppl. Figure 2, related to Fig 2: Expression of myeloid cells only**

(A) tSNE plot of myeloid cell sub-types after re-clustering the myeloid cells only (separated by conditions). Colored according to cellular identity.

(B) tSNE plot of different known myeloid cell markers.

(C) Dotplot representing the expression of few known markers of pDCs. The color of the dot represents the expression level, and the size of the dot represents the percentage of cells in each cluster expressing the gene.

**
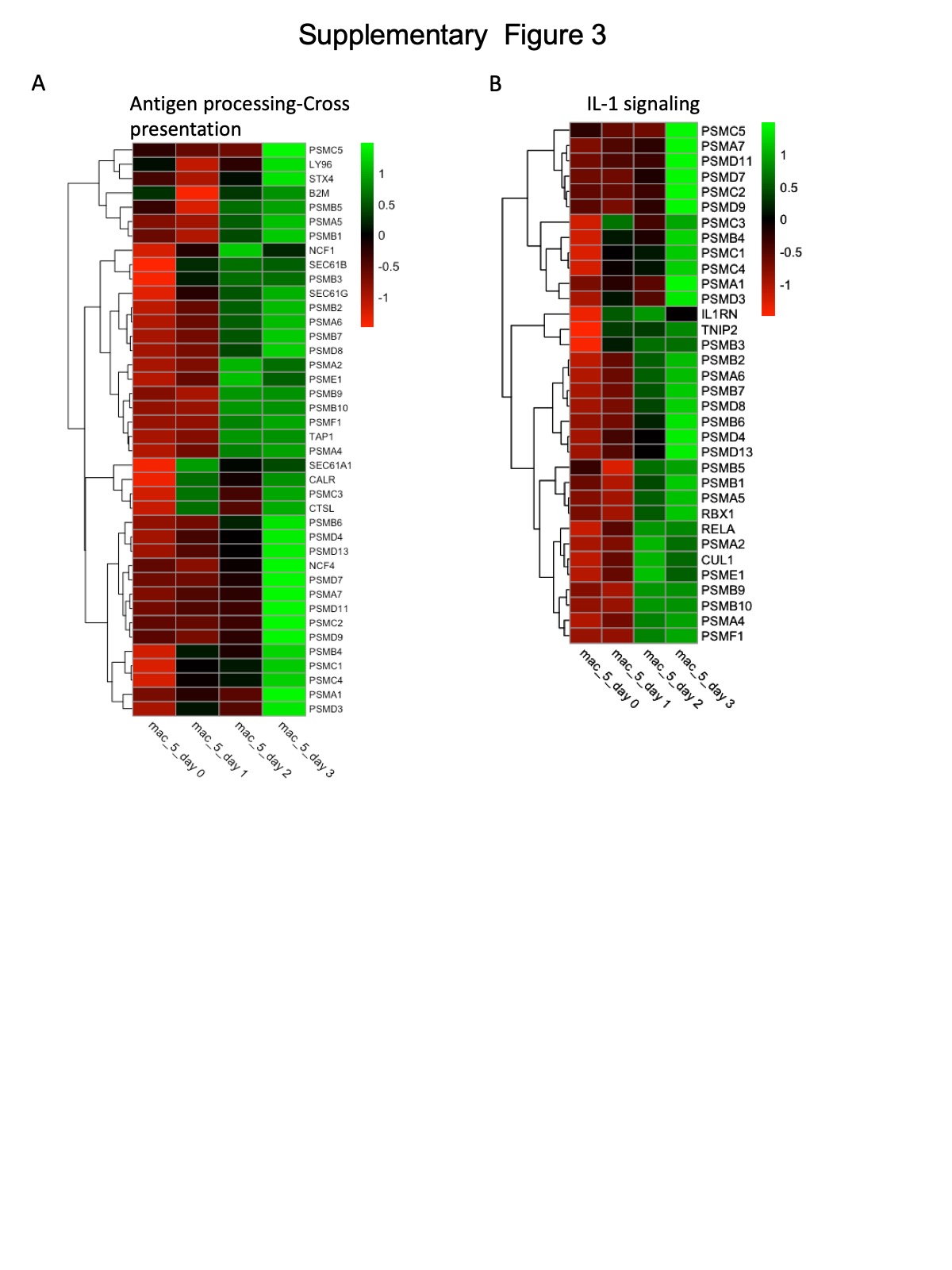
**

**Suppl. Figure 3, related to Fig 4: Expression of selected genes** **in cluster mac_5**

Heatmap with average normalized expression of the genes related to (A) Antigen processing-cross presentation pathway, and (B) IL-1 signaling pathway in different conditions (baseline, 1dpi, 2dpi, 3dpi) in cluster mac_5.

**
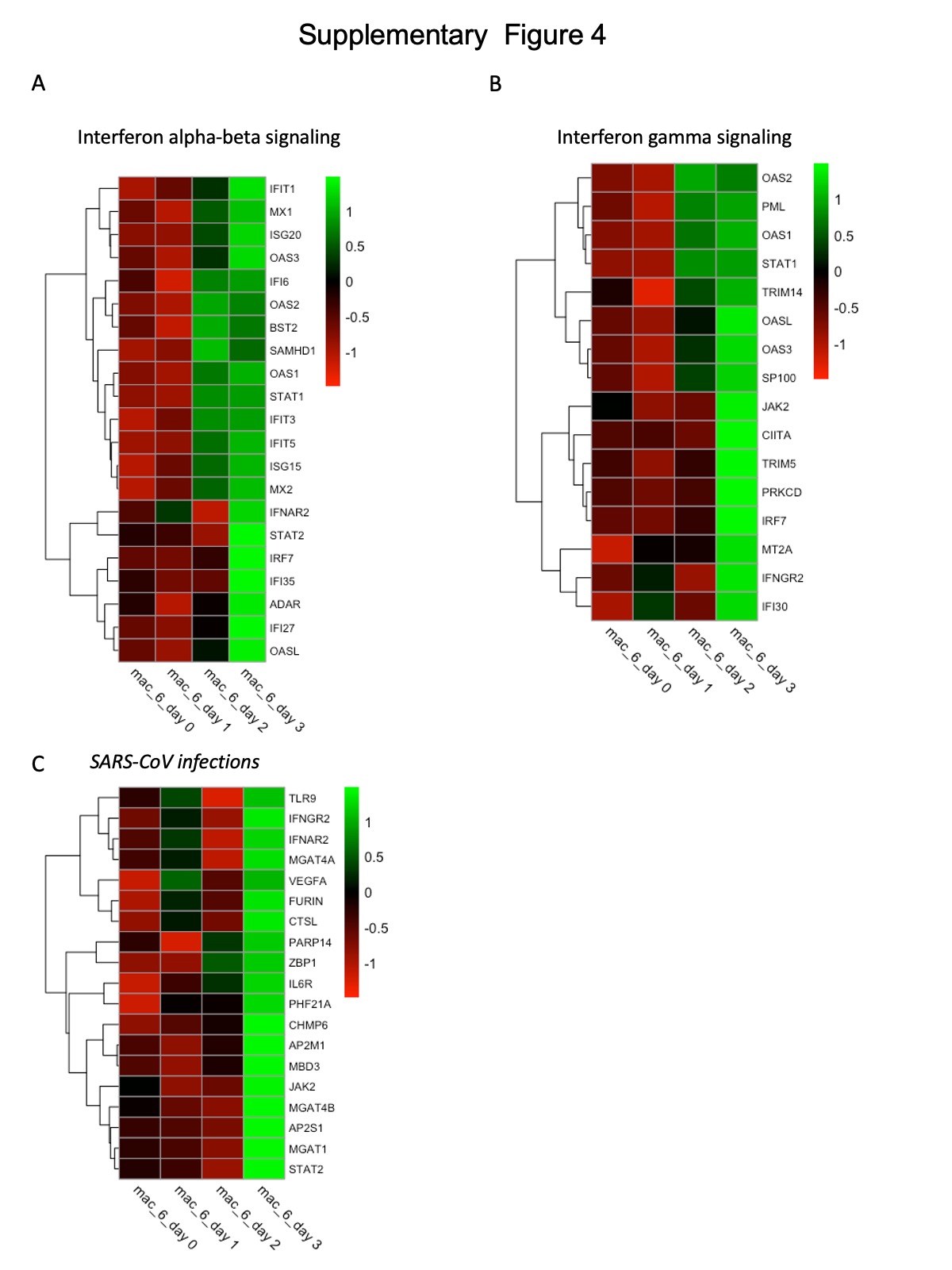
**

**Suppl. Figure 4, related to Fig 5: Expression of selected genes** **in cluster mac_6**

Heatmap with average normalized expression of the genes related to (A) IFN-α/β signaling, (B) IFN-γ signaling, and (C) SARS-CoV infections pathway in different conditions (baseline, 1dpi, 2dpi, 3dpi) in cluster mac_6.

**
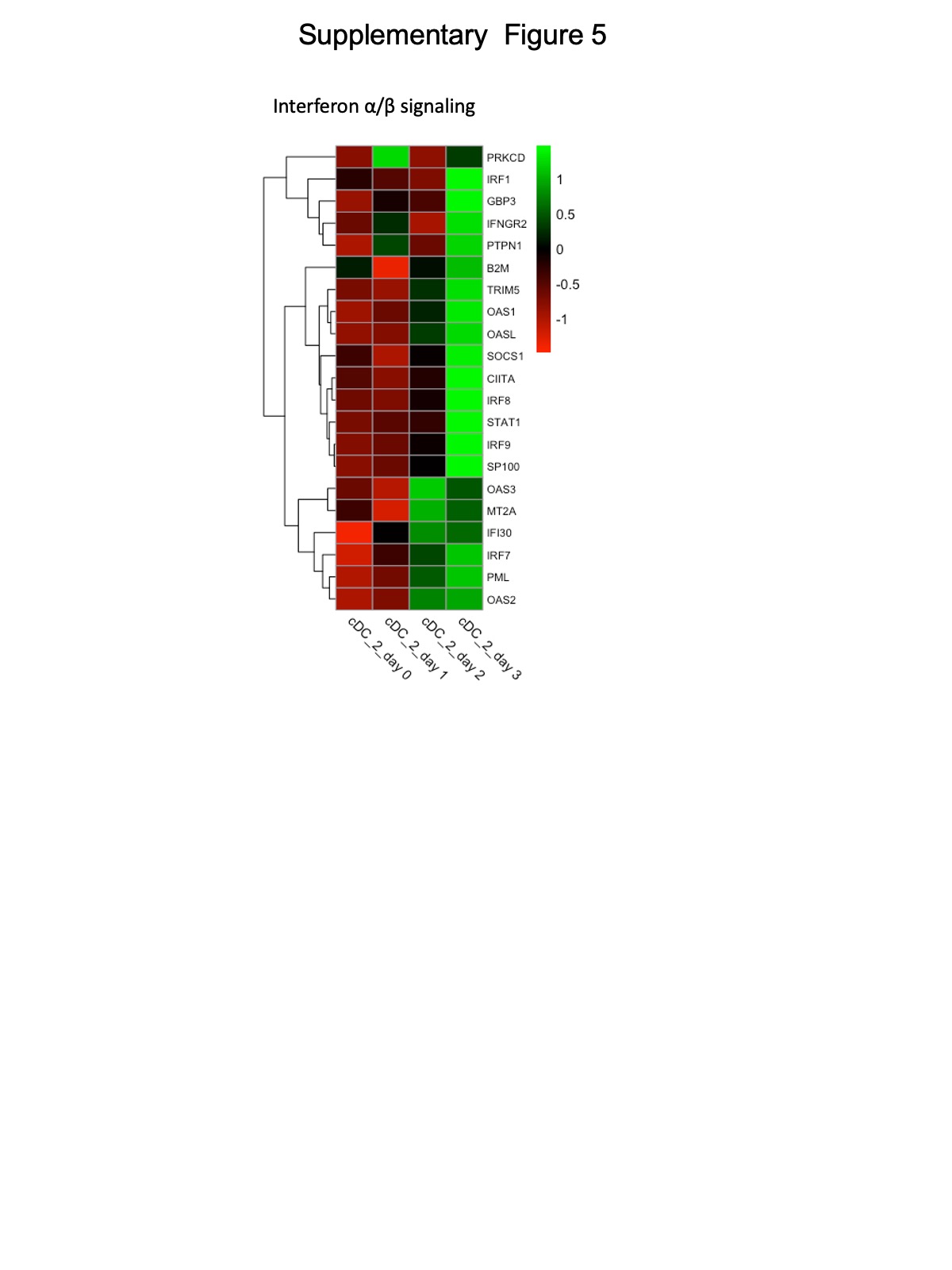
Suppl. Figure 5, related to Fig 10: Expression of selected genes** **in cluster cDC2**

Heatmap with average normalized expression of the genes related to (A) IFN-α/β signaling pathway in different conditions (baseline, 1dpi, 2dpi, 3dpi) in cluster cDC2.
